# Evidence mapping and meta-analysis of traditional Chinese medicine for postmenopausal osteoporosis

**DOI:** 10.1097/MD.0000000000050311

**Published:** 2026-08-28

**Authors:** Xi Xi, Xue Yan, Jingchun Fan, Yanying Zhang, Zhandong Wang

**Affiliations:** a First Clinical Medical College, Gansu University of Chinese Medicine, Lanzhou, China; b School of Public Health, Gansu University of Chinese Medicine, Lanzhou, China; c Gansu Provincial Experimental Animal Industry Technology Center, Lanzhou, China; d School of Integrated Traditional Chinese and Western Medicine, Gansu University of Chinese Medicine, Lanzhou, China.

**Keywords:** evidence map, meta-analysis, postmenopausal osteoporosis, traditional Chinese medicine

## Abstract

**Background:**

Postmenopausal osteoporosis (PMOP) is a common metabolic bone disorder characterized by estrogen deficiency – induced bone loss and increased fracture risk. Traditional Chinese medicine (TCM) has demonstrated unique advantages in PMOP management, but comprehensive, standardized evidence synthesis remains limited.

**Methods:**

Chinese and English databases (CNKI, Wanfang, VIP, SinoMed, PubMed, EMBASE, Web of Science, and Cochrane Library) were systematically searched up to July 31, 2024. Eligible studies included interventional, observational, and evidence-based research. Data extraction and quality assessment followed the Preferred Reporting Items for Systematic Reviews and Meta-Analyses 2020 statement. Meta-analyses were performed using RevMan 5.4 with random- or fixed-effects models depending on heterogeneity.

**Results:**

A total of 319 studies were included: 232 interventional, 60 observational, 25 systematic reviews or meta-analyses, and 2 guidelines. Among 99 randomized controlled trials, TCM interventions significantly improved clinical efficacy (risk ratio = 1.22, 95% confidence interval [CI]: 1.17–1.27, *I*^2^ = 55%) and reduced pain (standardized mean difference [SMD] = −2.15, 95% CI: −2.95 to − 2.17, *I*^2^ = 96%). TCM also enhanced bone mineral density at both lumbar (SMD = 0.74, 95% CI: 0.57–0.91, *I*^2^ = 90%) and femoral sites (SMD = 0.60, 95% CI: 0.41–0.80, *I*^2^ = 89%).

**Conclusion:**

TCM interventions significantly improve bone density and pain outcomes in PMOP. However, high heterogeneity and moderate methodological quality highlight the need for high-quality, standardized clinical trials integrating syndrome differentiation.

## 1. Introduction

Postmenopausal osteoporosis (PMOP) is a systemic skeletal disorder driven by ovarian functional decline and consequent estrogen deficiency, leading to deterioration of bone microarchitecture and loss of bone mass with reduced bone mineral density (BMD). It manifests clinically as pain, skeletal fragility, increased fracture risk, and psychosocial distress such as anxiety and depression.^[1]^ Recent epidemiological surveys in China report that osteoporosis affects approximately 32.1% of women aged over 50 and 51.6% of those over 65, roughly twice the prevalence in age-matched men, indicating a substantial health and socioeconomic burden.^[2]^

Current Western medical therapies – including estrogenic anabolic agents and bisphosphonates – effectively promote bone formation and inhibit resorption but are limited by long-term adverse effects, such as gastrointestinal irritation and atypical femoral fractures.^[3]^ Traditional Chinese medicine (TCM), with its long history of clinical use, regulates bone metabolism through multi-target mechanisms. Clinical evidence suggests that TCM interventions may improve BMD, enhance bone health, and inhibit bone resorption in patients with osteoporosis.^[4]^

However, most existing reviews focus on individual TCM formulas or single pharmacological components, lacking integrative visualization and standardized evaluation of overall evidence. To address these gaps, the present study combines evidence mapping with meta-analysis to comprehensively summarize the distribution, methodological quality, and therapeutic effects of TCM interventions for PMOP, providing an updated evidence base for further translational and clinical research.^[5,6]^

## 2. Methods

### 2.1. Literature search strategy

A comprehensive search was performed across 8 databases: CNKI, Wanfang, VIP, SinoMed, PubMed, EMBASE, Web of Science, and the Cochrane Library, covering the period from inception to July 31, 2024. The search strategy combined controlled vocabulary and free-text terms related to PMOP and TCM.

An example PubMed query was: (“traditional Chinese medicine” [Mesh] OR “Chinese herbal medicine” OR “Chinese patent medicine” OR TCM) AND (“postmenopausal osteoporosis” OR “osteoporosis, postmenopausal” [Mesh] OR PMOP)

Detailed search strings for each database are provided in Table S1, Supplemental Digital Content 1.

### 2.2. Inclusion criteria

Study design: Interventional studies (randomized controlled trials [RCTs], n-RCTs), observational studies (cohort, cross-sectional, and case–control), systematic reviews and meta-analyses, and clinical guidelines/consensus statements. Population: Women diagnosed with PMOP, postmenopausal status was defined as amenorrhea for ≥ 12 months.^[7]^

Intervention: TCM therapies in the treatment arm – herbal decoctions (e.g., Duhuo Jisheng Tang), patent medicines (e.g., Bushen Huoxue granules), single‐herb compounds (e.g., Epimedium and Eucommia), and other modalities (e.g., acupuncture). All interventions must adhere to relevant pharmacopeial or clinical guidelines.

Meta-analysis filtering: Only published RCTs; primary intervention must be TCM; control arm uses non-TCM treatment; comparisons include any form of TCM versus non-TCM, or TCM combined with a non-TCM modality versus the non-TCM modality alone; for multi-arm trials, only arms meeting these criteria are included; outcome measures must include BMD, overall response rate, and Visual Analog Scale (VAS) for pain.

### 2.3. Exclusion criteria

Studies were excluded if they: were duplicates, conference abstracts, or gray literature without peer review. Reported animal or in vitro experiments. Lacked retrievable full text or had incomplete or erroneous data. Focused solely on theoretical discussion, data mining, or pharmacological mechanisms without clinical outcomes. Failed to report treatment duration or menopausal diagnostic criteria.

### 2.4. Study selection and data extraction

After screening titles and abstracts against the inclusion/exclusion criteria, 2 investigators independently extracted data. A third reviewer adjudicated discrepancies; when necessary, original data sources were consulted and discussed to reach consensus.

### 2.5. Quality assessment

Risk of bias (RoB) was evaluated using the Cochrane Risk of Bias 2.0 (RoB 2.0) tool for RCTs, and A Measurement Tool to Assess Systematic Reviews (AMSTAR-2) and Appraisal of Guidelines for Research & Evaluation II (AGREE II) checklists for systematic reviews and guidelines, respectively.

A summary table and heatmap of bias assessments are presented in Table S2, Supplemental Digital Content 2.

### 2.6. Statistical analysis

The literature screening process was depicted using a flowchart. Data were presented in text, figures, and tables, including temporal trend line charts and bubble plots showing the distribution of outcome measures across TCM interventions. Evidence mapping was used to characterize the distribution of the available evidence according to study design, TCM intervention type, and outcome domain. Evidence was summarized using tables, temporal trend plots, and bubble plots to visualize research trends and the distribution of outcome measures. Analyses and graphics were generated using Microsoft Excel. For guidelines/consensus statements, intraclass correlation coefficients (ICC) were calculated using SPSS-AU to assess inter-rater consistency, with ICC > 0.80 indicating good agreement. Tables summarized the methodological quality of included systematic reviews/meta-analyses.

Subgroup analyses were conducted according to TCM intervention type, treatment duration, and sample size to explore potential sources of heterogeneity. Leave-one-out sensitivity analyses were performed by sequentially excluding individual studies to assess the robustness of pooled estimates. Publication bias was assessed using funnel plots and Egger tests when sufficient studies were available.

Meta-analyses were performed using Review Manager (RevMan) version 5.4 (Cochrane Collaboration). For dichotomous outcomes, risk ratios (RRs) with 95% confidence intervals (CIs) were calculated using the Mantel–Haenszel method. For continuous outcomes, standardized mean differences (SMDs) with 95% CIs were estimated using the DerSimonian–Laird random-effects model when heterogeneity (*I*^2^ > 50%) was present; otherwise, a fixed-effects model was applied. The pooled effect sizes and 95% CIs were presented as forest plots generated by RevMan.

## 3. Evidence map of TCM interventions for PMOP

### 3.1. Literature retrieval

**Figure 1.**
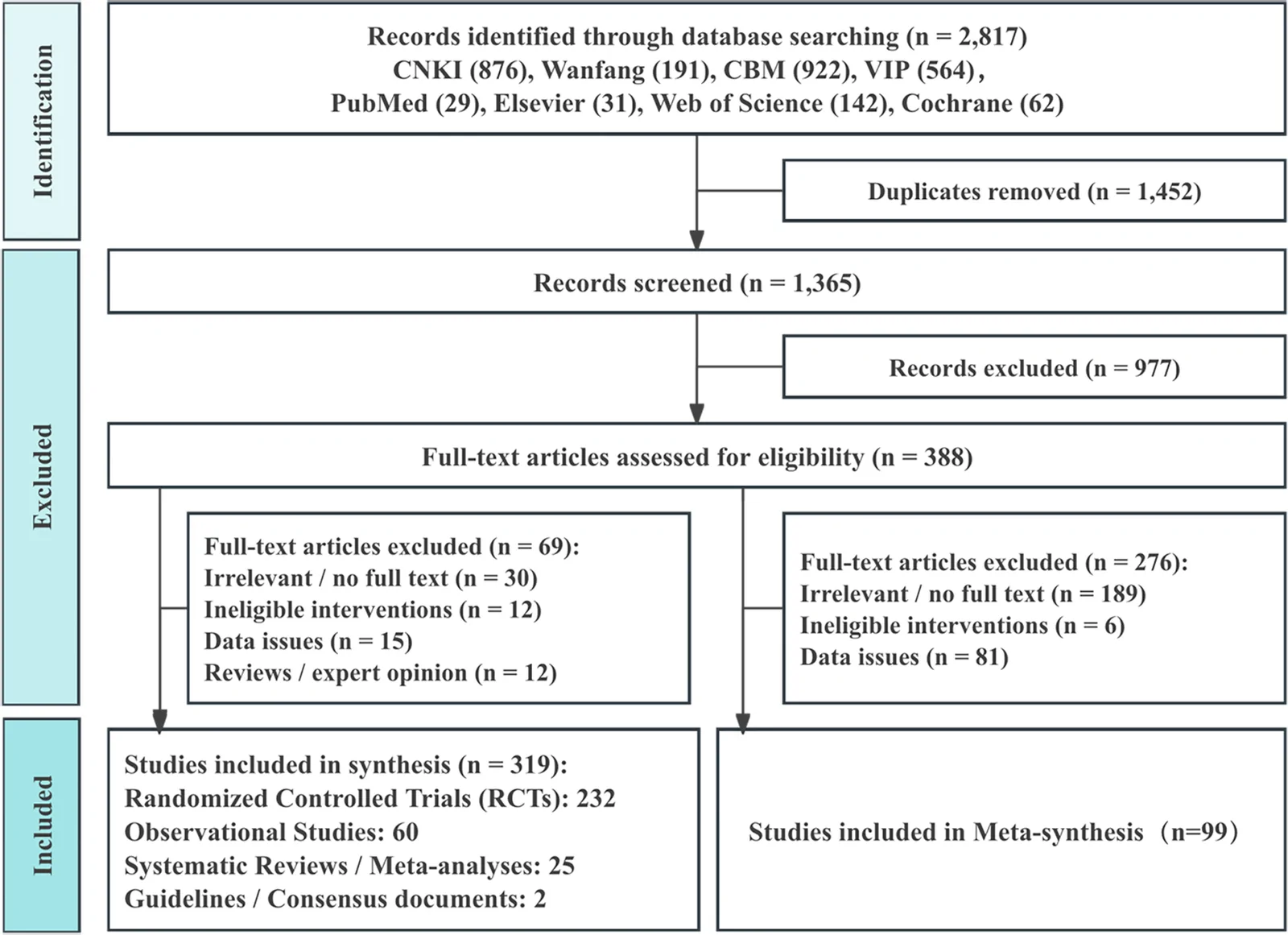
Article screening process. RCT = randomized controlled trial.

A total of 2817 records were identified through database searches. After removing duplicates and screening titles, abstracts, and full texts, 319 studies met the inclusion criteria, including 232 interventional studies (224 RCTs and 8 nRCTs), 60 observational studies, 25 systematic reviews or meta-analyses, and 2 clinical guidelines or consensus statements. A detailed selection process is illustrated in Figure 1. The evidence mapping component characterized the identified literature according to study design, intervention category, and outcome domain, providing an overview of the distribution and development of TCM research in PMOP.

### 3.2. Publication trends

**Figure 2.**
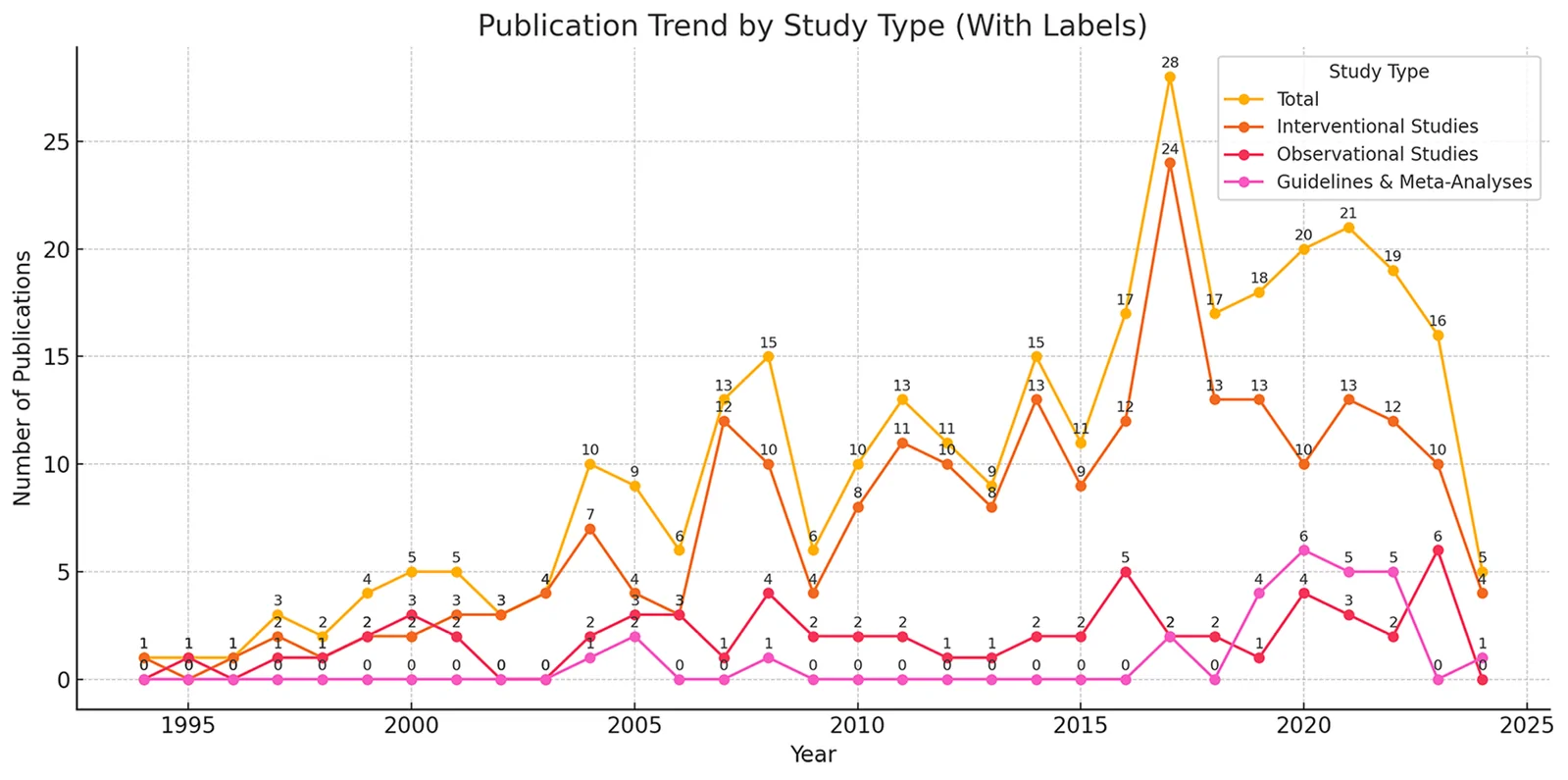
Trends in publications on TCM interventions for PMOP (1994–2024). PMOP = postmenopausal osteoporosis, TCM = traditional Chinese medicine.

Research on TCM interventions for PMOP has received growing academic attention, accompanied by a steady increase in publications over the past 3 decades. The number of clinical interventional trials, observational studies, and evidence‐based publications followed a similar upward trend. However, after reaching a peak in 2017, the annual output exhibited a mild decline – with minor fluctuations – through 2024 (Fig. 2). Between 1994 and 2024, a total of 311 publications on TCM interventions for PMOP were identified. The publication trajectory showed a gradual rise beginning in the early 2000s, a marked acceleration after 2014, and a peak output of 28 studies in 2017. Among these, interventional studies were predominant (n = 224) and have consistently contributed the largest proportion of publications since 2004. Observational studies accounted for 60 reports, maintaining a relatively stable presence, while guidelines and meta-analyses – emerging after 2004 – showed a moderate increase during the last decade, totaling 27 publications by 2024. Notably, the number of high-level evidence-based reports (e.g., meta-analyses and clinical guidelines) has risen since 2018, reflecting an increasing emphasis on evidence synthesis and methodological rigor in TCM research for PMOP. The apparent reduction in 2023 to 2024 may be attributed to indexing delays or evolving research priorities. Overall, these publication dynamics underscore the growing scientific recognition and methodological maturation of TCM-based approaches to PMOP, with interventional clinical trials remaining the dominant research design over the past 2 decades.

### 3.3. TCM treatment regimens

The interventions assessed in the included RCTs were classified into 4 major categories: herbal decoctions, Chinese patent medicines, other TCM modalities (e.g., acupoint application and acupuncture), and combination therapies (Table 1).

**Table 1:** Frequency distribution of different interventions.

Category	Intervention	Studies (n)	Percentage (%)
Herbal medicine	Decoctions	67	29.4
	Patent medicines	58	25.4
Other TCM methods	Acupuncture	6	2.6
	External application	3	1.3
	Qigong/Tai Chi	2	0.9
	Wax therapy	1	0.4
	Catgut embedding	1	0.4
	Fumigation	1	0.4
Combined therapies	TCM + Western medicine		
	Decoctions + Drugs	51	22.4
	Patent meds + Drugs	15	6.6
	Other TCM + Drugs	12	5.2
	TCM combinations		
	Decoctions + Qigong	3	1.3
	Decoctions + Acupunct.	2	1.3
	Other combinations*	6	2.6

* Includes: patent med combinations (1), acupuncture + application (1), herbal paste + fire needle (1), massage + herbs (1), patent meds + acupuncture (1), decoctions + fumigation (1).

TCM = traditional Chinese medicine.

Among these, herbal decoctions and patent medicines were the most frequently employed, whereas other TCM modalities were relatively uncommon. Overall, pure herbal interventions accounted for 54.8% of all RCTs (n = 125), including 67 trials on herbal decoctions and 58 trials on patent medicines. Combination regimens comprised 34.1% (78 integrated TCM–Western medicine studies) and 4.8% (11 multimodal TCM combination studies), while other TCM modalities represented 6.1% (14 studies).

Among the 78 combination-therapy trials, the most prevalent approach was herbal decoction combined with a Western drug (51 studies, 22.4%). For the 14 studies involving other TCM modalities, the interventions ranked by frequency were: comprehensive TCM therapy, acupuncture, topical application, qigong exercises, moxibustion wax therapy, buried-needle therapy, and fumigation.

### 3.4. Analysis of TCM preparations

**Figure 3.**
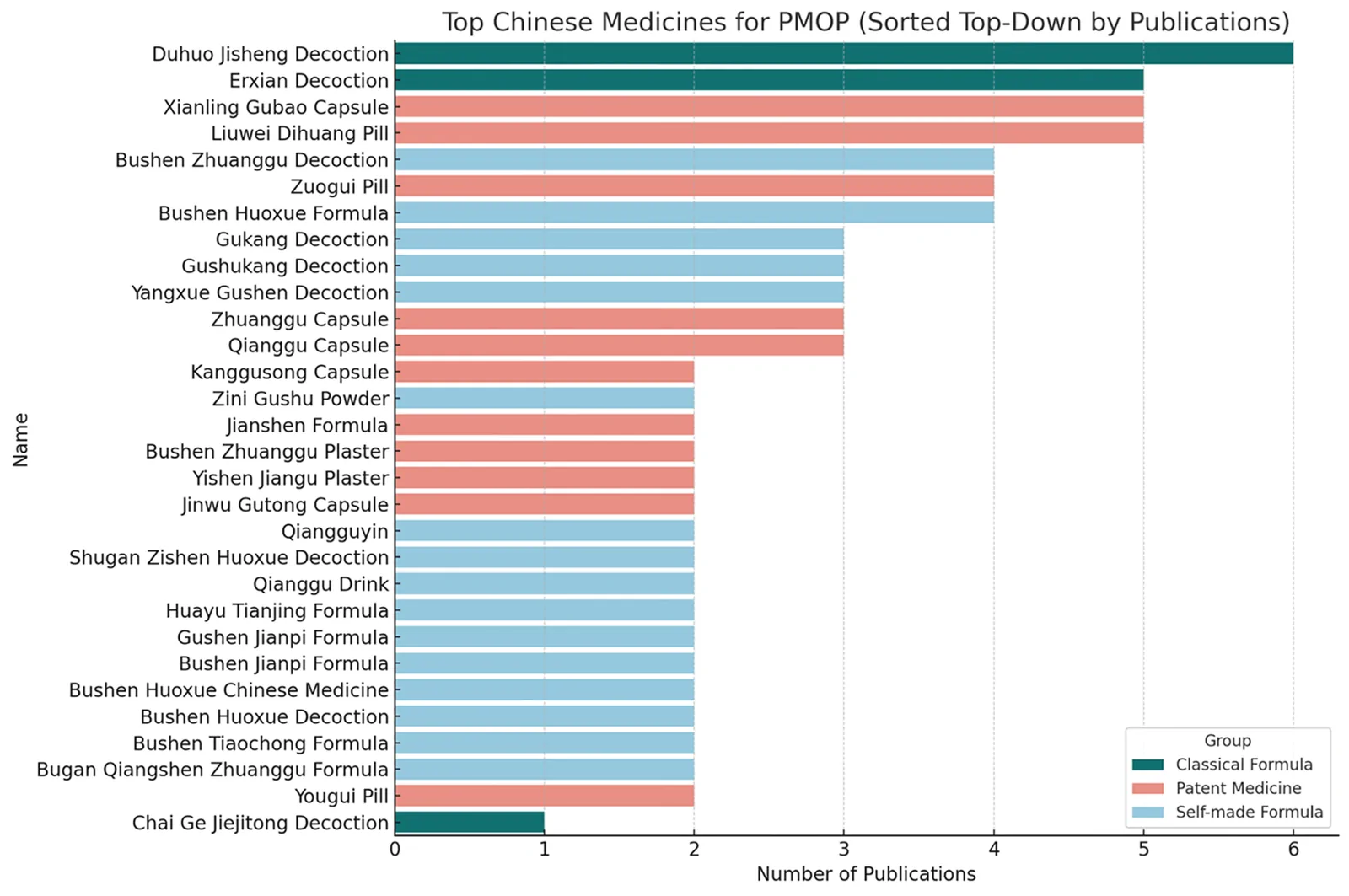
Number of published traditional Chinese materia medica. PMOP = postmenopausal osteoporosis.

The included RCTs evaluated both single and multimodality TCM interventions, encompassing herbal decoctions, Chinese patent medicines, and acupuncture-based therapies, among others. Herbal decoctions and patent medicines were predominant, whereas other modalities were comparatively less frequent. In total, 124 trials investigated 71 distinct herbal decoctions, among which the classic formulas Duhuo Jisheng Tang and Erxian Tang were most frequently employed. For clarity, Figure 3 lists only those decoctions evaluated in more than 1 trial. Similarly, 73 trials assessed 51 different Chinese patent medicines, with Liuwei Dihuang Wan and Xianling Gubao Capsules being the most commonly studied formulations (Fig. 3).

### 3.5. Distribution of TCM syndrome patterns

**Figure 4.**
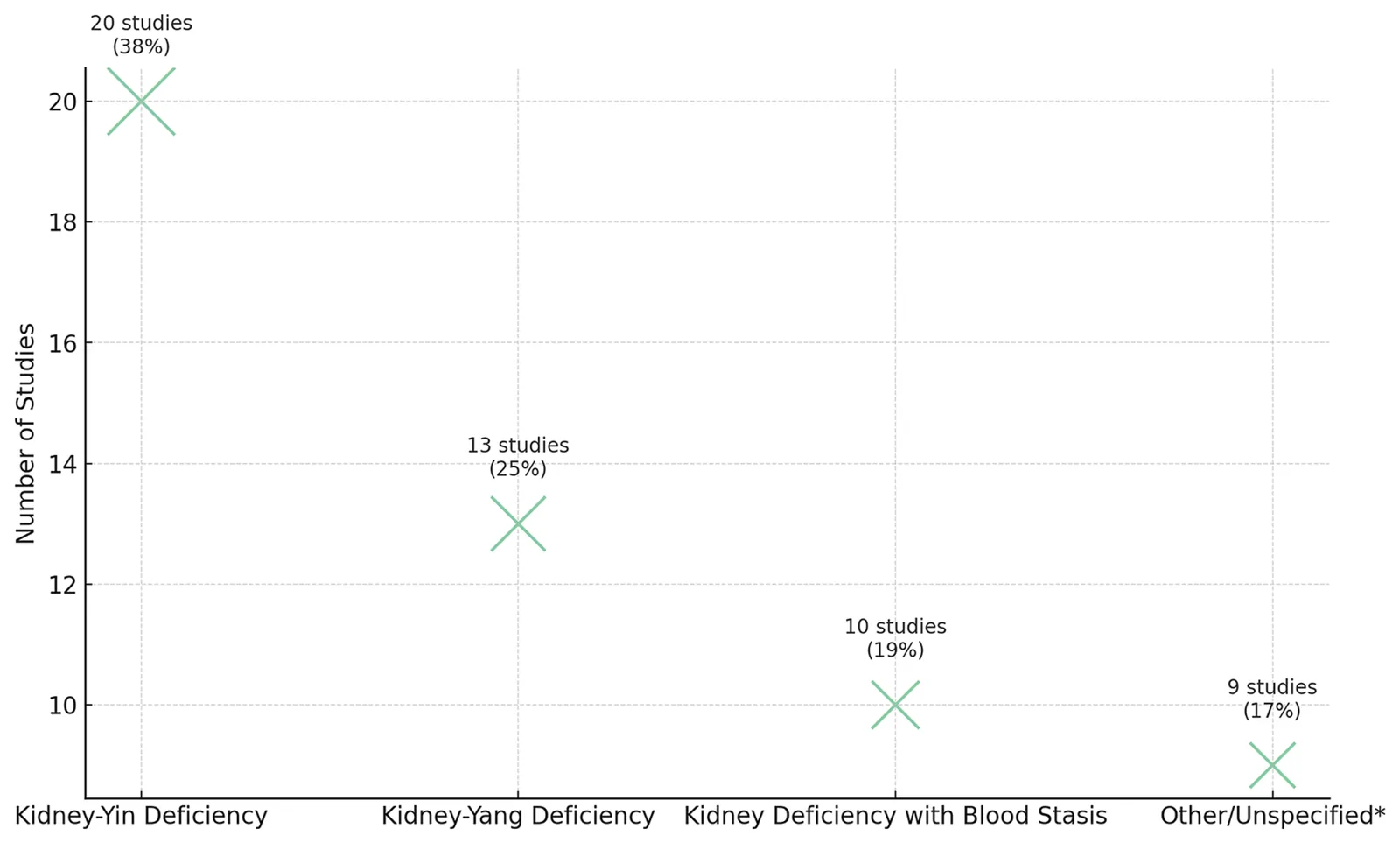
Distribution of traditional Chinese medicine (TCM) syndrome types in patients. *Includes: The syndrome was mentioned in the original study but not explicitly classified or was grouped under “other syndromes”.

A total of 52 studies reported TCM syndrome differentiation for patients with PMOP, encompassing 41 distinct intervention regimens. The identified syndromes were classified into 4 major categories: kidney-yin deficiency, kidney-yang deficiency, kidney deficiency with blood stasis, and unspecified or mixed patterns (syndrome mentioned but not clearly defined). Among these, kidney-yin deficiency was the most prevalent pattern (20 studies), followed by kidney-yang deficiency (13 studies; Fig. 4).

### 3.6. Outcome measure analysis

The outcome measures reported across the included RCTs were categorized into 7 major domains: clinical symptoms and rating scales, overall clinical efficacy, TCM-specific indices (such as syndrome-scale scores and syndrome efficacy), other observational markers (including adverse events, gut microbiota parameters, and nutritional indices), bone metabolism biomarkers, BMD, and hormonal or other biochemical indicators.

**Figure 5.**
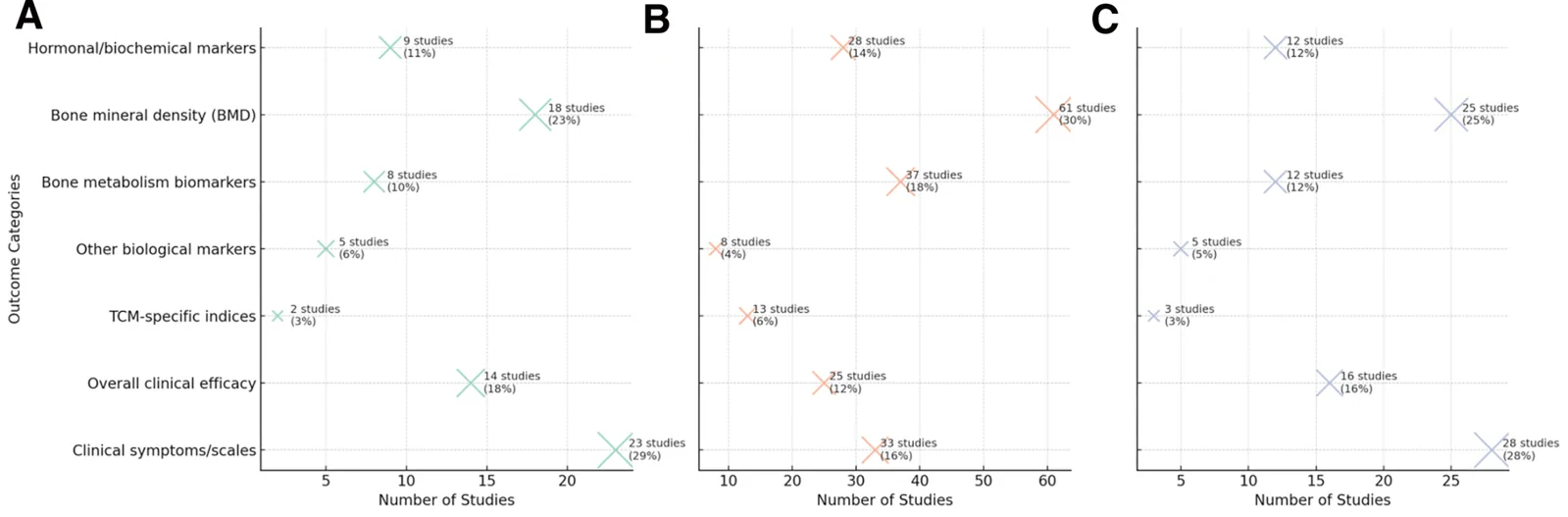
Evidence distribution of outcome indicator of different TCM in treatment of PMOP. Distribution of outcome measures among RCTs evaluating (A) TCM multimodality interventions, (B) Chinese patent medicines, and (C) other TCM modalities. BMD and clinical symptom improvement were the most frequently reported outcomes across all subgroups. BMD = bone mineral density, PMOP = postmenopausal osteoporosis, RCT = randomized controlled trial, TCM = traditional Chinese medicine.

Among the RCTs evaluating TCM multimodality interventions (34 studies involving 23 distinct regimens), outcome measures most frequently included clinical symptom and scale assessments (23 studies, 67.6%) and BMD measurements (18 studies, 52.9%), followed by overall clinical efficacy rates (14 studies, 41.2%), bone metabolism parameters (8 studies, 23.5%), and hormonal or biochemical indices (9 studies, 26.5%). Fewer trials assessed TCM-specific diagnostic indicators (2 studies, 5.9%) or other biological markers (5 studies, 14.7%; Fig. 5A).

For Chinese patent medicines (73 studies comprising 51 therapeutic regimens), the most commonly evaluated outcomes were BMD (61 studies, 83.6%) and bone metabolism parameters (37 studies, 50.7%), followed by clinical symptoms and scale assessments (33 studies, 45.2%) and overall clinical efficacy rates (25 studies, 34.2%). TCM-specific diagnostic indicators were reported in 13 studies (17.8%), while hormonal or biochemical indices and other biological markers were described in 28 (38.4%) and 8 (11.0%) studies, respectively (Fig. 5B).

Additionally, other TCM modalities (35 studies) primarily reported improvements in clinical symptoms and rating scales (28 studies, 80.0%) and BMD (25 studies, 71.4%), followed by clinical effectiveness rates (16 studies, 45.7%), bone metabolism–related markers (12 studies, 34.3%), and hormonal or biochemical parameters (12 studies, 34.3%). Only a few studies assessed TCM pattern differentiation indicators (3 studies, 8.6%) or other observational parameters (5 studies, 14.3%; Fig. 5C).

### 3.7. RoB assessment (Cochrane Tool)

**Figure 6.**
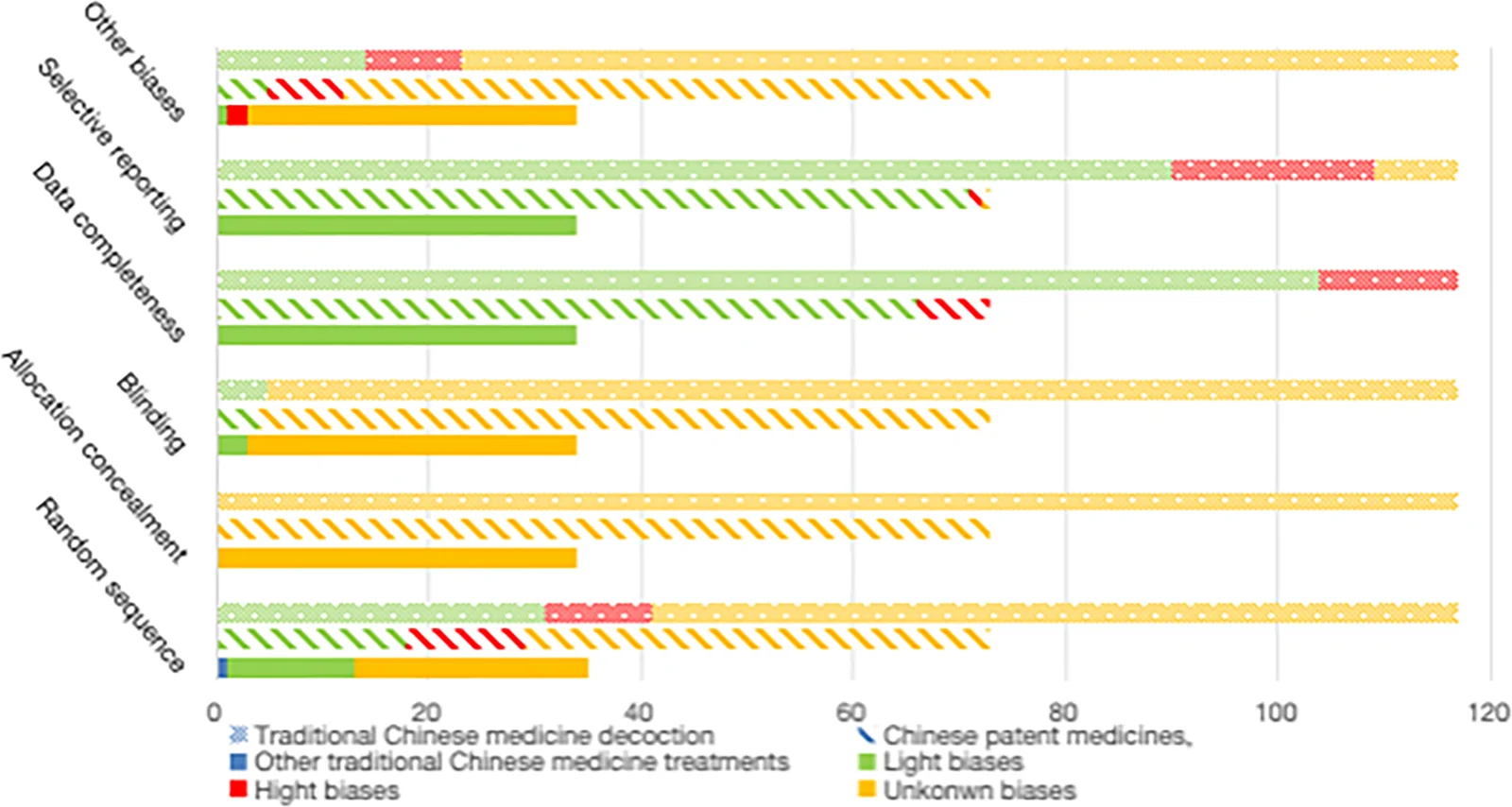
RCT methodology quality evaluation. RCT = randomized controlled trial.

All included RCTs exhibited varying degrees of unclear or high RoB (Fig. 6). For studies involving Chinese herbal decoctions, the primary sources of bias were insufficient reporting of allocation concealment, absence of participant or assessor blinding, and other unspecified methodological deficiencies. High RoB was most commonly attributed to uncertainties in random sequence generation, selective outcome reporting, and other unclarified biases, whereas low risk was mainly associated with adequate randomization procedures and complete outcome data.

In studies investigating Chinese patent medicines, a similar pattern was observed. The unclear risk primarily resulted from incomplete descriptions of allocation concealment and blinding, while high risk stemmed from poorly defined randomization methods and incomplete data reporting. Conversely, low risk was observed in reporting transparency and outcome completeness.

For trials assessing other TCM modalities (e.g., acupuncture and external applications), the unclear risk predominantly arose from the lack of detailed information on randomization, allocation concealment, and blinding. In contrast, low risk was mostly identified in data integrity and selective reporting domains. Overall, the methodological quality of the included studies was suboptimal, underscoring the need for greater adherence to CONSORT and Preferred Reporting Items for Systematic Reviews and Meta-Analyses (PRISMA) reporting standards and for rigorous trial design in future TCM clinical research.

### 3.8. Methodological quality of systematic reviews/meta-analyses

The methodological quality of the 25 included systematic reviews/meta-analyses was evaluated using the AMSTAR checklist (total score: 11 points). The quality scores ranged from 4 to 11 points, with: 15 studies^[8–22]^ demonstrating validated methodological rigor (score ≥ 7); 10 studies^[23–32]^ suggesting potential therapeutic effects but with methodological limitations (score < 7).

### 3.9. Methodological quality of clinical guidelines/consensus statements

Two TCM clinical guidelines for PMOP management^[33,34]^ (both China-based) were assessed using the AGREE II instrument: Inter-rater reliability: Excellent consistency (ICC = 0.947, >0.80 threshold); Domain scores (mean standardized percentages): Scope/purpose: 93.11%, Clarity: 88.89%, Stakeholder involvement: 87.50%, Rigor of development: 82.74%, Applicability: 37.50%, and Editorial independence: 16.67%. Overall rating: Grade B (moderate quality).

## 4. Meta-analysis of TCM for PMOP

**Figure 7.**
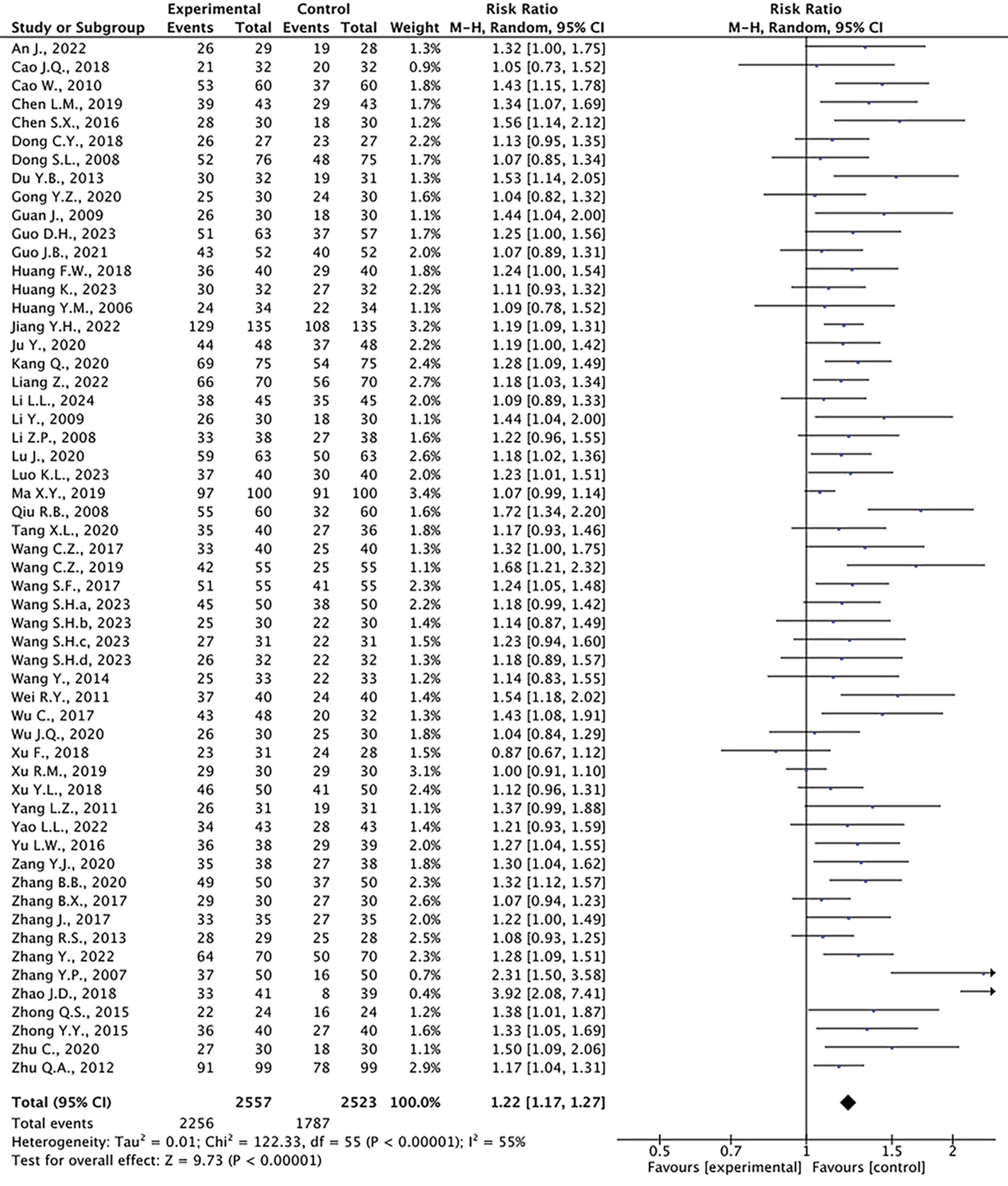
Forest plot of the effect of TCM compound group and control on effective rate (n = 5080, *I*^2^ = 55%, random effect model). CI = confidence interval, TCM = traditional Chinese medicine.

**Figure 8.**
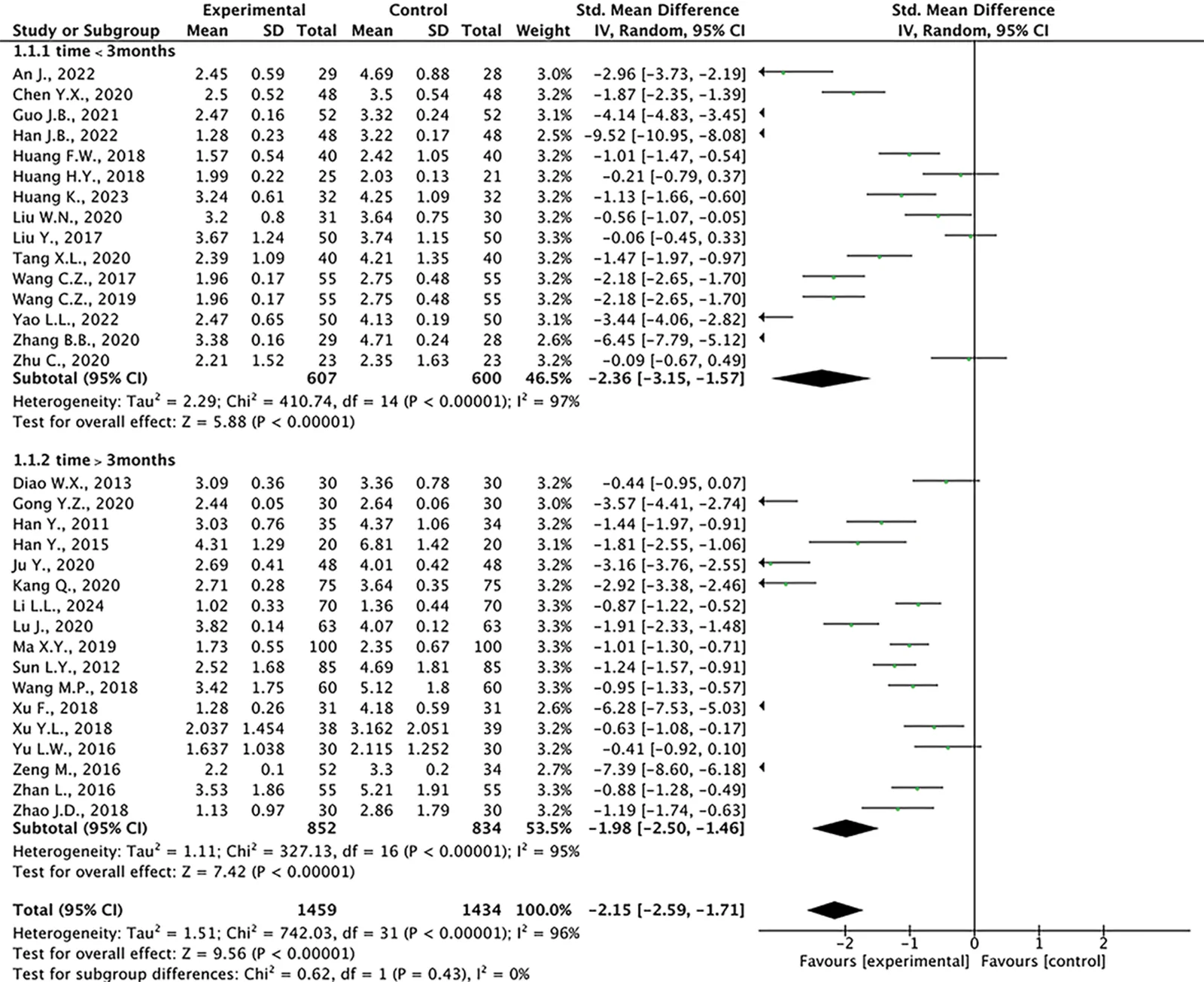
Forest plot of the effect of TCM compound group and control on VAS (n = 2893, *I*^2^ = 95%, random effect model). CI = confidence interval, TCM = traditional Chinese medicine, VAS = Visual Analog Scale.

**Figure 9.**
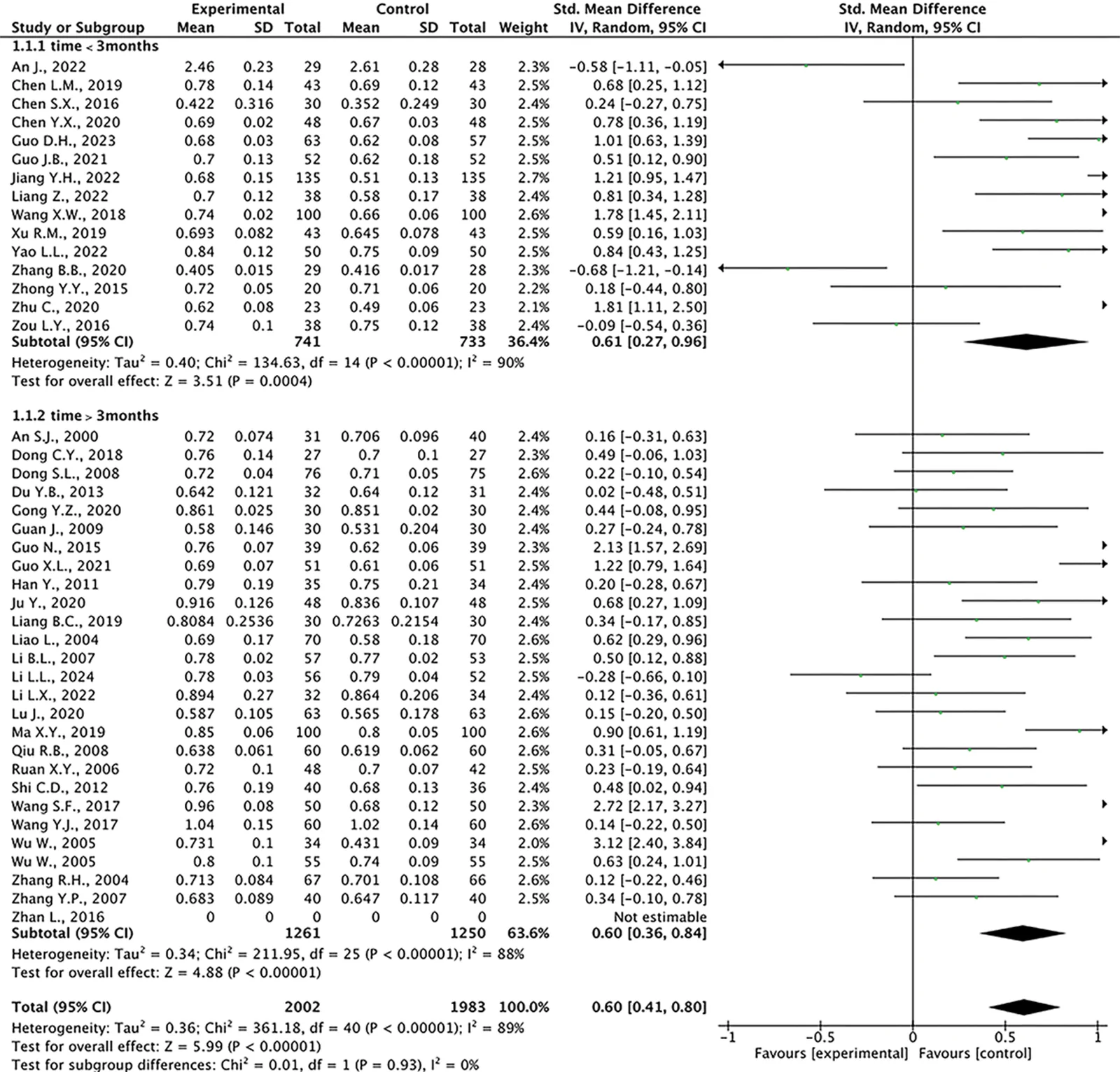
Forest plot of the effect of TCM compound group and control on F-BMD (n = 3925, *I*^2^ = 89%, random effect model). CI = confidence interval, F-BMD = F-bone mineral density, TCM = traditional Chinese medicine.

**Figure 10.**
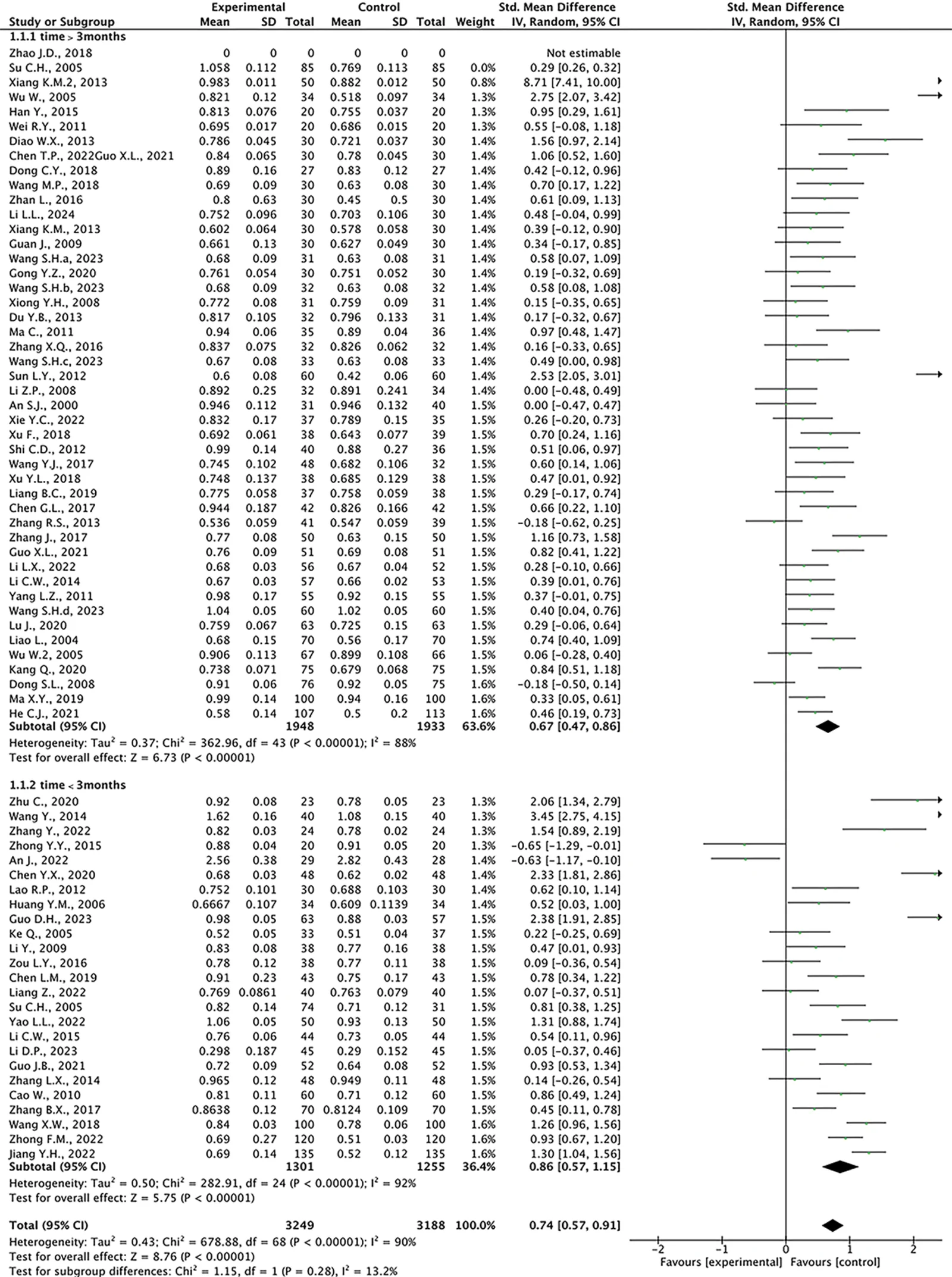
Forest plot of the effect of TCM compound group and control on V-BMD (n = 6437, *I*^2^ = 90%, random effect model). CI = confidence interval, V-BMD = V-bone mineral density, TCM = traditional Chinese medicine.

A total of 99 RCTs were included in the meta-analysis, confirming the overall therapeutic efficacy of TCM for PMOP (Fig. 1). The pooled analysis demonstrated significant improvements in both BMD and pain outcomes (Table 2). The overall clinical response rate was 88.9% in TCM groups versus 71.7% in control groups (RR = 1.22, 95% CI: 1.17–1.27; *I*^2^ = 55%), indicating a clinically meaningful benefit (Fig. 7). Although moderate heterogeneity was detected, the effect direction consistently favored TCM. Subgroup analyses suggested that intervention type and treatment duration may partially contribute to the observed heterogeneity. Integrated TCM–Western medicine interventions and multi-component herbal decoctions generally showed larger effect estimates than single patent medicines, while treatment durations ≥ 6 months tended to be associated with more consistent improvements in BMD and pain outcomes. Sensitivity analyses using sequential exclusion of individual studies did not materially alter the pooled estimates, supporting the robustness of the overall findings. Regarding pain outcomes, the meta-analysis showed that TCM interventions significantly reduced VAS pain scores (SMD = −2.15, 95% CI: −2.95 to −2.17, *P* < .00001; Mantel–Haenszel random-effects model), confirming a robust analgesic effect (Fig. 8). However, substantial heterogeneity was observed (*I*^2^ = 96%), suggesting considerable variation among trials in treatment duration, formula composition, and assessment methods. For bone density outcomes, TCM significantly improved femoral BMD compared with control treatments (SMD = 0.60, 95% CI: 0.41–0.80, *P* < .00001; *I*^2^ = 89%), and lumbar BMD (SMD = 0.74, 95% CI: 0.57–0.91, *P* < .00001; *I*^2^ = 90%). Despite the high heterogeneity, sensitivity analysis confirmed the stability of the pooled estimates, and the CIs remained narrow, supporting the robustness of the findings (Figs. 9 and 10).

**Table 2:** Summary of meta-analysis results.

Outcome	No. of studies	Model	Effect size	95% CI	*I*^2^ (%)
Clinical effective rate	59	Random	RR = 1.22	1.17 to 1.27	55
VAS pain score	32	Random	SMD = −2.15	−2.95 to −2.17	96
Lumbar BMD	41	Random	SMD = 0.74	0.57 to 0.91	90
Femoral BMD	70	Random	SMD = 0.6	0.41 to 0.80	89

BMD = bone mineral density, CI = confidence interval, RR = risk ratio, SMD = standardized mean difference, VAS = Visual Analog Scale.

**Figure 11.**
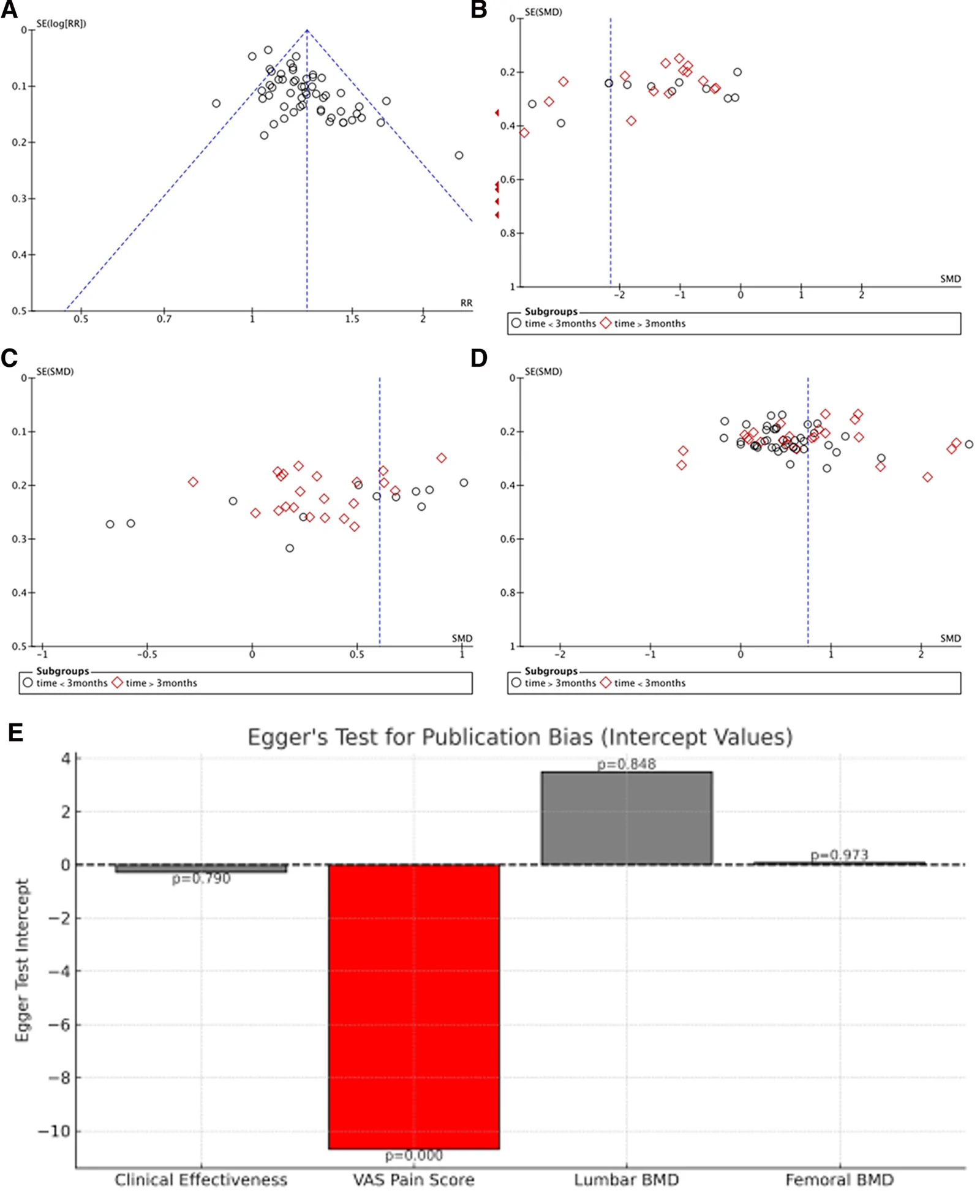
Funnel plots for publication bias across 4 outcomes: (A) overall clinical efficacy, (B) VAS score, (C) femoral BMD, and (D) lumbar BMD. Significant asymmetry was observed for VAS (Egger test *P* < .01), whereas other outcomes demonstrated low risk of publication bias. BMD = bone mineral density, VAS = Visual Analog Scale.

Collectively, these results indicate that TCM interventions provide consistent benefits in pain relief and bone density improvement, supporting their potential role as effective adjunctive therapies for PMOP. To evaluate the risk of publication bias among the studies included in this systematic review, funnel plots were constructed for 4 primary outcome measures – overall clinical efficacy, VAS score, femoral BMD, and lumbar BMD – and were supplemented by *Egger* test for quantitative verification (Fig. 11). The funnel plot for overall clinical efficacy exhibited good symmetry, with a balanced distribution of studies on both sides of the vertical axis, indicating a low risk of publication bias. In contrast, the funnel plot for VAS score displayed a notable leftward asymmetry, characterized by an absence of small-sample studies on the lower right side, suggesting substantial publication bias. This observation was further supported by *Egger* test (*P* < .01), confirming the statistical significance of the bias. For femoral BMD, the funnel plot demonstrated mild leftward asymmetry, suggesting a possible minor publication bias; *Egger* test yielded borderline significance (*P* ≈ .05), consistent with this finding. By contrast, the funnel plot for lumbar (vertebral) BMD showed overall symmetry, and Egger test revealed no significant bias (*P* > .05), indicating a low publication bias risk for this outcome.

## 5. Discussion

### 5.1. Characteristics of literature on TCM in the treatment of PMOP

PMOP does not have an exact equivalent term in TCM but is generally classified under syndromes such as “bone wilting” (骨痿) and “bone impediment” (骨痹).^[35]^ Although publication activity in this field has fluctuated, a recent decline has been observed, with only 10 studies published in 2024, likely reflecting delays in ongoing research and indexing processes. Since 2016, research topics have become increasingly diversified, encompassing mechanistic investigations, novel TCM modalities, and integrative therapeutic approaches. By 2020, the discipline entered a phase of evidence-based consolidation, characterized by an increasing number of systematic reviews and meta-analyses that quantitatively evaluate the efficacy of TCM interventions. For instance, a systematic review published in 2025 assessed the efficacy of acupuncture for primary osteoporosis and reported significant improvements in BMD (MD = 0.04, 95% CI: 0.03–0.06, *P* < .001) and overall clinical efficacy rate (RR = 1.24, 95% CI: 1.14–1.34, *P* < .001).^[36]^

Despite the growing body of evidence supporting the efficacy of TCM for PMOP, the overall methodological rigor of available studies remains moderate.^[36]^ Many RCTs lack adequate reporting of randomization procedures, allocation concealment, and blinding, resulting in a substantial proportion of studies categorized as having an “unclear” RoB. This observation is consistent with recent findings indicating limited adherence to rigorous reporting standards in TCM clinical trial protocols, with an average SPIRIT-TCM compliance rate of 35.4%.^[37]^ Currently, Chinese herbal decoctions and patent medicines are the most frequently utilized modalities. Whether administered as monotherapy or in combination with Western medicine, these approaches have demonstrated significant therapeutic benefits in alleviating clinical symptoms and minimizing treatment-related adverse effects. In addition, non-pharmacological TCM therapies, such as herbal steaming-washing and moxibustion, have also shown verified effectiveness and favorable safety profiles.^[38]^

In total, 71 classical formulas and 51 types of Chinese patent medicines have been documented in clinical studies. Among these, the classical prescriptions Liuwei Dihuang Pill, Duhuo Jisheng Decoction, and Erxian Decoction were the most frequently utilized. In addition, numerous self-designed herbal formulas have been applied with favorable clinical outcomes.

These prescriptions primarily act by tonifying the kidney, consistent with TCM theory, which attributes PMOP to kidney essence deficiency, liver dysfunction, and blood stasis obstructing the meridians.^[39]^ However, clinical research incorporating TCM syndrome differentiation remains limited and often lacks diagnostic precision. Among the few studies that addressed syndromic patterns, kidney-yin deficiency was the most frequently identified type. Only 2 trials implemented comparative interventions based on differentiated syndromes,^[40,41]^ indicating that syndrome differentiation – a core principle of TCM – has not been adequately integrated into modern clinical research. Furthermore, TCM-specific outcome measures, such as syndrome score indices, are infrequently reported, which does not adequately reflect the theoretical and diagnostic strengths of TCM. In summary, although TCM provides promising therapeutic options for PMOP, existing literature is characterized by methodological heterogeneity, insufficient standardization, and limited use of syndrome-based diagnostic approaches. Addressing these limitations will be essential for establishing high-quality evidence and advancing the integration of TCM into global osteoporosis management.

In clinical research, outcome measures are fundamental for evaluating therapeutic efficacy and ensuring the validity of evidence.^[42]^ However, studies investigating TCM interventions for PMOP have revealed several methodological deficiencies in this regard. Most notably, the commonly reported “total effective rate” often lacks integration with broader, patient-centered indicators such as pain severity, functional mobility, nutritional status, and quality of life – all of which are essential for a comprehensive assessment of treatment outcomes. Furthermore, the economic burden associated with PMOP treatment is considerable, yet this dimension is seldom incorporated into existing outcome evaluations. Globally, the annual cost of osteoporotic fractures is estimated to exceed USD 5 trillion, with North America and Europe accounting for a major share of total healthcare expenditures.^[43]^ Recognizing the growing importance of cost-effectiveness, the National Healthcare Security Administration of China formally integrated pharmacoeconomic evaluation into its national reimbursement negotiation framework in 2022.^[44]^ Despite this policy advancement, few clinical studies on PMOP have included economic endpoints, such as cost-benefit or cost-utility analyses. This gap underscores an urgent need for future TCM-related trials to incorporate health economic evaluation within study design. Doing so would enhance the real-world applicability of TCM interventions and provide evidence-based support for healthcare decision-makers and insurance systems in optimizing long-term osteoporosis management strategies.

### 5.2. Methodological quality of TCM research on PMOP

The appraisal of methodological quality is a critical component in the process of evidence generation.^[45]^ An analysis of 224 RCTs investigating TCM treatments for PMOP, conducted using the Cochrane Risk of Bias tool, revealed that the overall quality of evidence remains suboptimal. A predominant limitation among these studies was incomplete or insufficient methodological reporting. In many cases, essential trial information – such as baseline participant characteristics, methods of randomization and blinding, handling of withdrawals or dropouts, sample size calculations, and disclosure of potential conflicts of interest – was either inadequately described or entirely omitted. The absence of these methodological details may compromise the reliability, reproducibility, and interpretability of research findings. Notably, previous meta-epidemiological investigations have demonstrated that inadequate or unreported allocation concealment and blinding can lead to an overestimation of treatment effects by approximately 30 to 50%.^[46]^ This underscores the urgent need for stricter adherence to international reporting guidelines, such as CONSORT and SPIRIT-TCM, to enhance the credibility and transparency of future TCM clinical trials.

Methodological weaknesses such as selection bias, information bias, and confounding may further undermine the reliability of existing findings. These methodological limitations may also influence the pooled estimates by exaggerating treatment effects or increasing uncertainty across studies. Therefore, the statistically significant pooled effects observed in this meta-analysis should be interpreted cautiously in light of the overall RoB and the substantial heterogeneity among included trials. To enhance the quality and clinical relevance of studies on TCM for PMOP, several methodological improvements are recommended. First, transparent and comprehensive reporting should be ensured throughout study design, implementation, data analysis, and interpretation to enhance reproducibility and scientific rigor. Second, adequate sample sizes and longer follow-up durations are needed to minimize bias and strengthen external validity. Third, outcome assessments should reflect the multitarget nature of TCM interventions rather than relying solely on composite “effective rate” indicators. Fourth, funding sources and potential conflicts of interest must be clearly disclosed to reduce publication bias. Fifth, investigator training and multidisciplinary collaboration should be reinforced, integrating expertise in evidence-based medicine, pharmacology, and clinical practice to improve both methodological rigor and translational impact.

Furthermore, the AGREE II instrument and AMSTAR tool were employed to appraise the methodological quality of relevant clinical guidelines and systematic reviews. The included guidelines were generally rated as Grade B, with notable limitations including unclear contributor roles, limited multidisciplinary participation, and insufficient reporting of implementation challenges. Future development of TCM-related clinical guidelines and evidence syntheses should strictly follow international reporting frameworks to improve transparency and completeness. The PRISMA Statement provides essential standards for conducting and reporting systematic reviews and meta-analyses^[47]^ while the AGREE II tool offers a structured approach for developing and evaluating clinical guidelines or expert consensus documents. Adherence to these standards will enhance methodological rigor, transparency, and credibility, thereby reducing bias and strengthening the reliability of clinical evidence in TCM research on PMOP.

### 5.3. Discussion of meta-analysis

These findings collectively demonstrate that TCM interventions confer significant benefits across multiple clinical and physiological outcomes in patients with PMOP. In terms of clinical efficacy, TCM treatment markedly improved the overall response rate, reflecting its advantages in holistic regulation and pattern-based (syndrome differentiation) therapy, and underscoring its therapeutic relevance in osteoporosis management. Regarding pain relief, the substantial reduction in VAS scores indicates potential analgesic effects of TCM, likely associated with its ability to modulate inflammatory responses, improve microcirculation, and enhance qi and blood flow. However, pain perception remains inherently subjective, and methodological variability – including differences in evaluation timing, treatment duration, and formulation composition – may partially account for the observed heterogeneity. With respect to BMD, TCM interventions demonstrated significant improvements in both femoral and lumbar sites, lending further support to the TCM principle of “tonifying the kidney and strengthening the bone.” Although substantial heterogeneity was present, the consistent direction of effect across studies suggests that TCM may regulate bone metabolism through multifaceted and systemic mechanisms, addressing the complex pathophysiology of osteoporosis at multiple biological levels.

Although moderate-to-high heterogeneity (*I^2^* = 55–96%) was observed across the pooled analyses, the overall direction of effect remained consistent, supporting the robustness and reliability of the findings. Subgroup analyses suggested that intervention type and treatment duration may partially contribute to the observed heterogeneity. Integrated TCM–Western medicine interventions and multi-component herbal decoctions generally showed larger effect estimates than single patent medicines, while treatment durations ≥ 6 months tended to be associated with more consistent improvements in BMD and pain outcomes. Leave-one-out sensitivity analyses did not materially alter the pooled estimates, supporting the robustness of the overall findings. The observed heterogeneity likely reflects differences in herbal formulations, intervention combinations, treatment duration, syndrome differentiation, patient characteristics, and outcome assessment methods. These sources of clinical heterogeneity are intrinsic to TCM clinical research and may limit the direct comparability of interventions across studies. Future research should adopt standardized diagnostic criteria, uniform outcome measures, and large-scale multicenter trial designs to minimize between-study variation and strengthen evidence reliability.

Regarding publication bias, most outcomes were not substantially affected by small-study effects or selective reporting. However, the VAS pain outcome domain exhibited a clear asymmetry in funnel plots, indicating a potential publication bias, possibly due to preferential publication of positive pain-relief findings from smaller or less rigorous trials. This bias underscores the importance of prospective trial registration, comprehensive outcome reporting regardless of result, and strict adherence to PRISMA and CONSORT guidelines. Incorporating unpublished studies, preprints, and negative results in future analyses would further mitigate bias and improve the transparency of TCM evidence synthesis.

### 5.4. Clinical implications and future directions

In contemporary clinical practice, TCM interventions for PMOP are frequently administered in combination with conventional pharmacologic or rehabilitative therapies, reflecting their synergistic and integrative role within comprehensive treatment frameworks. However, most of the included studies were conducted within limited geographic regions, potentially restricting the generalizability and external validity of their findings. Future research should therefore include populations from diverse ethnic and healthcare backgrounds to better represent the global applicability of TCM interventions.

Moreover, syndrome differentiation – a cornerstone of TCM diagnosis and treatment – was inadequately implemented in most existing clinical trials. Upcoming studies should aim to incorporate standardized syndrome classification and individualized treatment protocols grounded in TCM theoretical principles. In parallel, the integration of modern clinical assessment tools, including measures of symptom severity, quality of life, functional capacity, and psychological well-being, would enable a more objective and multidimensional evaluation of treatment outcomes.

Overall, there is a pressing need to improve both the methodological quality and the scientific depth of TCM-related clinical research on PMOP. The implementation of rigorous trial designs, standardized outcome measures, and transparent reporting practices will enhance the credibility and reproducibility of findings, ultimately facilitating the broader clinical acceptance and international integration of TCM in the management of osteoporosis.

## 6. Summary

In recent years, several meta-analyses have evaluated the efficacy of individual TCM formulas or patent medicines in PMOP; however, most focused on single interventions, lacked visualized evidence mapping, and did not comprehensively assess the methodological quality of the included studies. Furthermore, few studies have integrated data from both Chinese and English sources to explore global research trends. To address these gaps, the present study combines evidence mapping with meta-analysis to provide a comprehensive overview of TCM interventions for PMOP. This approach allows visualization of research distribution, identification of methodological shortcomings, and quantitative synthesis of efficacy indicators.

## 7. Strengths and limitations

This study has several strengths. First, it combined evidence mapping with quantitative meta-analysis to characterize both the distribution and pooled effects of TCM interventions for PMOP. Second, multiple evidence types and methodological quality domains were systematically evaluated. Third, both Chinese and international databases were searched to provide a broad overview of the available evidence. Several limitations should also be considered. First, substantial heterogeneity was observed across several pooled outcomes, which may reflect differences in TCM interventions, treatment duration, patient characteristics, comparators, and outcome assessment. Second, many included RCTs had unclear or high RoB, particularly regarding randomization, allocation concealment, and blinding. Third, potential publication bias was observed for some outcomes. Finally, most included studies were conducted in China, which may limit the generalizability of the findings to other healthcare settings.

This systematic review and meta-analysis followed the PRISMA 2020 guidelines. The study was not prospectively registered in PROSPERO, as it was based on retrospective evidence mapping of published data.

## Author contributions

**Data curation:** Xi Xi, Xue Yan.

**Funding acquisition:** Yanying Zhang.

**Methodology:** Xi Xi, Jingchun Fan.

**Project administration:** Zhandong Wang.

**Software:** Xi Xi.

**Supervision:** Jingchun Fan, Yanying Zhang, Zhandong Wang.

**Visualization:** Xue Yan.

**Writing – original draft:** Xi Xi.

**Writing – review & editing:** Xi Xi.




