# Supplementary material for: Evidence mapping and meta-analysis of traditional Chinese medicine for postmenopausal osteoporosis
Source: Medicine (Baltimore). 2026 Aug 28;105(35):e50311. doi: 10.1097/MD.0000000000050311 (PMC13529136; doi:10.1097/MD.0000000000050311)
Supplement: Supplementary file 1 [file medi-105-e50311-s001.docx]

| Chinese database | | |
| --- | --- | --- |
| CNKI | n=876 | (Topic: Traditional Chinese Medicine + Chinese herbal medicine) OR (Keywords/Abstract: Traditional Chinese Medicine + Chinese herbal medicine (exact match)) OR (Topic: Chinese herbal medicine) OR (Keywords/Abstract: Chinese herbal medicine (exact match)) AND (Topic: Postmenopausal osteoporosis + Postmenopausal osteoporotic fracture + Postmenopausal osteoporotic bone loss) OR (Keywords/Abstract: Postmenopausal osteoporosis + Postmenopausal osteoporotic fracture + Postmenopausal osteoporotic bone loss (exact match)) |
| wanfang | n=191 | (Title or Keywords: (Traditional Chinese Medicine) or Keywords: (Traditional Chinese Medicine) or Title or Keywords: (Traditional Chinese Medicine) or Abstract: (Traditional Chinese Medicine)) and (Title or Keywords: (Postmenopausal Osteoporosis) or Abstract: (Postmenopausal Osteoporosis) or Title or Keywords: (Postmenopausal Osteoporosis Fracture) or Abstract: (Postmenopausal Osteoporosis Fracture) or Title or Keywords: (Postmenopausal Osteoporosis Bone Mass Loss) or Abstract: (Postmenopausal Osteoporosis Bone Mass Loss)) |
| VIP | n=922 | (M= Traditional Chinese medicine OR U= Traditional Chinese medicine OR M= Traditional Chinese medicine OR U= traditional Chinese medicine) AND (M= Postmenopausal osteoporosis OR R= postmenopausal osteoporosis OR M= postmenopausal osteoporosis fracture OR U= postmenopausal osteoporosis fracture OR M= postmenopausal osteoporosis bone mass loss OR U= Postmenopausal osteoporosis Bone mass loss |
| CBM | n=564 | "Postmenopausal Osteoporosis "[Title: Smart] OR" Postmenopausal Osteoporosis "[Abstract: Smart] OR "Postmenopausal osteoporotic Fractures "[Title: Smart] OR" Postmenopausal osteoporotic Fractures "[Abstract: Smart] OR "Postmenopausal osteoporotic bone mass loss "[Title: Smart] OR "In postmenopausal osteoporosis bone mass loss" [abstract: smart]) AND (" Chinese medicine "[title: intelligent] OR" Chinese medicine "[abstract: smart] OR" Chinese medicine "[title: intelligent] OR" Chinese medicine "[abstract: smart]) |
| English database | | |
| PubMed | n=29 | #1"chinese herbal drug"[Title/Abstract] OR "traditional chinese drug"[Title/Abstract] OR "chinese patent drug"[Title/Abstract] OR "chinese traditional medicine"[Title/Abstract] OR "TCM"[Title/Abstract]  #2"postmenopausal osteoporosis"[Title/Abstract] OR "PMOP"[Title/Abstract]  #3: #1 AND #2/("chinese herbal drug"[Title/Abstract] OR "traditional chinese drug"[Title/Abstract] OR "chinese patent drug"[Title/Abstract] OR "chinese traditional medicine"[Title/Abstract] OR "TCM"[Title/Abstract]) AND ("postmenopausal osteoporosis"[Title/Abstract] OR "PMOP"[Title/Abstract]) |
| Embase | n=31 | Title, abstract, keywords: (Chinese herbal drug OR traditional Chinese drug OR Chinese patent drug OR Chinese traditional medicine OR TCM) AND (postmenopausal osteoporosis OR PMOP) |
| WOS | n=142 | #1 (((((TS=(PMOP)) OR TI=(PMOP)) OR AB=(PMOP)) OR AB=(postmenopausal osteoporosis)) OR TS=(postmenopausal osteoporosis)) OR TI=(postmenopausal osteoporosis)  #2 ((((((((((((((AK=(chinese herbal drug)) OR TI=(chinese herbal drug)) OR AB=(chinese herbal drug)) OR AB=(traditional chinese drug)) OR AK=(traditional chinese drug)) OR AB=(traditional chinese drug)) OR AB=(chinese patent drug)) OR AK=(chinese patent drug)) OR TI=(chinese patent drug)) OR TI=(chinese traditional medicine)) OR AB=(chinese traditional medicine)) OR AK=(chinese traditional medicine)) OR AK=(TCM)) OR TI=(TCM)) OR AB=(TCM)  #3 :#2 AND #1 |
| Cochrane | n=62 | #1 (Chinese herbal drug):ti,ab,kw OR (traditional Chinese drug):ti,ab,kw OR (TCM):ti,ab,kw OR (Chinese patent drug):ti,ab,kw AND (Chinese traditional medicine):ti,ab,kw (Word variations have been searched)  #2 (postmenopausal osteoporosis):ti,ab,kw OR (PMOP):ti,ab,kw (Word variations have been searched)  #3 #1 AND #2 |
