# Supplementary material for: Evidence mapping and meta-analysis of traditional Chinese medicine for postmenopausal osteoporosis
Source: Medicine (Baltimore). 2026 Aug 28;105(35):e50311. doi: 10.1097/MD.0000000000050311 (PMC13529136; doi:10.1097/MD.0000000000050311)
Supplement: Supplementary file 2 [file medi-105-e50311-s002.docx]

| **Chinese patent medicine-RCT** | | | | | | | | | | | | |  |
| --- | --- | --- | --- | --- | --- | --- | --- | --- | --- | --- | --- | --- | --- |
| **Year** | **Author** | | **Formula** | | **Category** | **Intervention Type** | **Random Sequence** | **Allocation Concealment** | **Blinding** | **Data Integrity** | **Selective Reporting** | **Other Bias** |  |
| 2022 | Liu YI | | Zuogui Pill | | Blank | Chinese patent medicine | yes | unclear | unclear | yes | no | unclear |  |
| 2006 | Ruan XIANGYAN | | Qianggu Capsule | | Estrogen | Chinese patent medicine | yes | unclear | unclear | yes | no | unclear |  |
| 2013 | Tan YAMING | | He-gu Granule | | Calcium preparation | Chinese patent medicine | yes | unclear | unclear | yes | no | unclear |  |
| 2023 | An JUAN | | Jingang Pill | | Western medicine | Chinese patent medicine | yes | unclear | unclear | yes | no | unclear |  |
| 2022 | Liang ZHUANG | | Yougui Pill + Acupuncture | | Western medicine | Chinese + Traditional | yes | unclear | unclear | yes | no | unclear |  |
| 2022 | Xie LIHUA | | Xuling Jiangu Granule | | Western medicine | Chinese patent medicine | unclear | unclear | unclear | yes | no | unclear |  |
| 2021 | Sun TONGHUA | | Liqi Guben Group | | Western medicine | Chinese patent medicine | unclear | unclear | unclear | no | no | unclear |  |
| 2020 | Liu WEINAN | | Yishen Jiangu Ointment | | Western medicine | Chinese + Western | unclear | unclear | unclear | yes | no | unclear |  |
| 2020 | Lu JUN | | Zuogui Pill | | Western medicine | Chinese + Western | unclear | unclear | unclear | yes | no | unclear |  |
| 2020 | Kang QING | | Zuogui Pill | | Calcium preparation | Chinese patent medicine | unclear | unclear | unclear | yes | no | unclear |  |
| 2020 | Zhang BINGBIN | | Guwei Decoction | | Xianling Gubao Capsule | Chinese patent medicine | unclear | unclear | unclear | yes | no | unclear |  |
| 2020 | Zang YOUJUN | | Zhuanggupian | | Calcium preparation | Chinese patent medicine | unclear | unclear | unclear | yes | yes | unclear |  |
| 2019 | Ma XIAOYONG | | Xianling Gubao Capsule | | Western medicine | Chinese + Western | no | unclear | unclear | yes | no | unclear |  |
| 2019 | Liang BOCHENG | | Qianggu Plaster | | Western medicine | Chinese patent medicine | yes | unclear | unclear | yes | no | no |  |
| 2019 | Liao XINBO | | Bushen Zhuanggu Plaster | | Western medicine | Chinese patent medicine | unclear | unclear | unclear | yes | no | unclear |  |
| 2018 | Xu YULU | | Jinggu Bugugu Granule | | Western medicine | Chinese patent medicine | yes | unclear | unclear | yes | no | no |  |
| 2018 | Cao JUNQING | | Yougui Pill | | Western medicine | Chinese + Western | unclear | unclear | unclear | yes | no | no |  |
| 2018 | Zhao JINDONG | | Fufang Bushen Huoxue Granule | | Xianling Gubao Capsule | Chinese patent medicine | yes | unclear | unclear | yes | no | unclear |  |
| 2017 | Zhang JIAN | | Gushu Kang | | Western medicine | Chinese + Western | yes | unclear | yes | yes | no | unclear |  |
| 2017 | Wang DANHUI | | Luha Mixture | | Xianling Gubao Capsule | Chinese patent medicine | unclear | unclear | unclear | yes | no | unclear |  |
| 2016 | Dai YINAN | | Yinyanghuo Capsule | | Blank | Chinese patent medicine | unclear | unclear | unclear | yes | no | unclear |  |
| 2016 | Zhang XIAOQIAN | | Niuyin Mixture | | Western medicine | Chinese + Western | unclear | unclear | unclear | yes | no | unclear |  |
| 2016 | Zeng MING | | Jintian Ge Capsule | | Western medicine | Chinese patent medicine | unclear | unclear | unclear | yes | no | unclear |  |
| 2016 | Yu LANWEI | | Yishen Jiangu Ointment | | Western medicine | Chinese + Western | no | unclear | unclear | no | no | unclear |  |
| 2015 | Li CHUNWEN | | Qianggu Drink Granule | | Western medicine | Chinese + Western | unclear | unclear | unclear | yes | no | unclear |  |
| 2015 | Zhong YINYAN | | Xianling Gubao Capsule | | Western medicine | Chinese + Western | unclear | unclear | unclear | yes | no | unclear |  |
| 2014 | Zhao XUEYUAN | | Rongjin Tablet + Pulse Therapy | | Western medicine | Chinese + Western | unclear | unclear | unclear | no | no | unclear |  |
| 2014 | Li TAO | | Liuwei Dihuang Pill | | Western medicine | Chinese patent medicine | yes | unclear | unclear | yes | no | unclear |  |
| 2013 | Du YIBIN | | Gushu Granule | | Western medicine | Chinese patent medicine | unclear | unclear | unclear | yes | no | unclear |  |
| 2013 | Guo DEHUA | | Duzhong–Xuduan | | Western medicine | Chinese + Western | yes | unclear | unclear | yes | no | unclear |  |
| 2023 | Han JUNBU | | Zhuangguzhitong Capsule | | Blank | Chinese patent medicine | yes | unclear | unclear | yes | no | unclear |  |
| 2022 | Xie YUCHENG | | Bushen Huoxue Decoction | | Western medicine | Chinese + Western | no | unclear | unclear | yes | no | unclear |  |
| 2015 | Zhang YUN | | Liuwei Dihuang Pill | | Western medicine | Chinese + Western | yes | unclear | unclear | yes | no | unclear |  |
| 2015 | Cui MIN | | Shuangdiao Zhuanggu Treasure | | Western medicine | Chinese patent medicine | unclear | unclear | unclear | no | no | yes |  |
| 2014 | Dang XIAOLING | | Lugu Yishen Miguling | | Calcium preparation | Chinese patent medicine | unclear | unclear | unclear | no | no | yes |  |
| 2013 | Li BAOLIN | | Jian Shen Formula | | Calcium preparation | Chinese patent medicine | unclear | unclear | unclear | yes | no | unclear |  |
| 2012 | Sun LANying | | Guyu Ling Capsule | | Calcium preparation | Chinese patent medicine | unclear | unclear | unclear | yes | no | unclear |  |
| 2007 | Wang JIAMOU | | Gushu Tong | | Gushu Kang | Chinese patent medicine | unclear | unclear | unclear | yes | unclear | unclear |  |
| 2005 | Su CHENGHAI | | Zhuanggugu Capsule | | Calcium preparation | Chinese patent medicine | no | unclear | unclear | yes | no | unclear |  |
| 2005 | Li SHUANGLEI | | Zhuanggugu Capsule | | Calcium preparation | Chinese patent medicine | yes | unclear | unclear | yes | no | unclear |  |
| 2004 | Zhang RONGHUA | | Yigu Capsule | | Western medicine | Chinese patent medicine | yes | unclear | yes | yes | no | no |  |
| 2012 | Shi CHUANDAO | | Kangshu Jiangu Granule | | Calcium preparation | Chinese patent medicine | unclear | unclear | unclear | yes | no | unclear |  |
| 2011 | Ma CHUN | | Liuwei Dihuang Pill | | Calcium preparation | Chinese patent medicine | unclear | unclear | unclear | yes | no | unclear |  |
| 2011 | Wei RONGYING | | Kanggusong Capsule | | Calcium preparation | Chinese patent medicine | unclear | unclear | unclear | yes | no | no |  |
| 2011 | Wu ZHONGQIN | | Xianling Gubao Capsule | | Calcium preparation | Chinese patent medicine | unclear | unclear | unclear | yes | no | unclear |  |
| 2010 | Wan LEI | | Shiwei Gukang Oral Liquid | | Western medicine | Chinese patent medicine | unclear | unclear | unclear | yes | no | unclear |  |
| 2009 | Wang JINTAI | | Jinwu Gutong Capsule | | Western medicine | Chinese patent medicine | unclear | unclear | unclear | yes | no | unclear |  |
| 2008 | Zhang SUIJIAN | | Qidan Dihuang Powder | | Qianggu Capsule | Chinese patent medicine | no | unclear | unclear | yes | no | no |  |
| 2008 | Wen CHAO | | Zhuanggugu Capsule | | Qianggu Capsule | Chinese patent medicine | unclear | unclear | unclear | yes | no | unclear |  |
| 2008 | Li ZHONGPING | | Kanggusong Capsule | | Western medicine | Chinese patent medicine | no | unclear | unclear | no | no | yes |  |
| 2008 | Miao JIANQI | | Self-made Formula | | Calcium preparation | Chinese patent medicine | unclear | unclear | unclear | yes | no | unclear |  |
| 2008 | Li BAOLIN | | Jian Shen Formula | | Western medicine | Chinese patent medicine | unclear | unclear | unclear | yes | no | unclear |  |
| 2006 | Xia WENFANG | | Qianggu Capsule | | Western medicine | Chinese patent medicine | unclear | unclear | unclear | yes | no | unclear |  |
| 2006 | Li ZHIYING | | Xianling Gusong Capsule | | Calcium preparation | Chinese patent medicine | unclear | unclear | unclear | yes | no | unclear |  |
| 2004 | Deng WEIMIN | | Bushen Zhuanggu Powder | | Western medicine | Chinese patent medicine | unclear | unclear | unclear | yes | no | unclear |  |
| 2003 | Shao MIN | | Gukang | | Blank | Chinese patent medicine | unclear | unclear | unclear | yes | no | unclear |  |
| 2000 | Chen JIANPING | | Gukang | | Western medicine | Chinese patent medicine | unclear | unclear | unclear | yes | no | unclear |  |
| 2024 | Gu HONGFEI | | Artificial Tiger Bone Capsule | | Western medicine | Chinese + Western | unclear | unclear | unclear | yes | no | unclear |  |
| 2009 | Guan JIAN | | Liuwei Dihuang Pill | | Calcium preparation | Chinese patent medicine | unclear | unclear | unclear | no | no | unclear |  |
| 2004 | Zhao GUANG | | Qianggu Capsule | | Western medicine | Chinese patent medicine | no | unclear | unclear | yes | no | unclear |  |
| 2007 | Zheng WENKUI | | Jinwu Gutong Capsule | | Calcium preparation | Chinese patent medicine | yes | unclear | yes | yes | no | no |  |
| 2022 | Li LUNXIN | | Liuwei Dihuang Pill | | Blank | Chinese patent medicine | yes | unclear | unclear | yes | no | unclear |  |
| 2017 | Lin KANG | | Xianling Gubao Capsule | | Western medicine | Chinese + Western | unclear | unclear | unclear | yes | no | unclear |  |
| 2005 | Wu WEN | | Xianling Gubao Capsule | | Calcium preparation | Chinese patent medicine | unclear | unclear | unclear | yes | no | unclear |  |
| 2008 | Yin TAO | | Gushu Ling Capsule | | Blank | Chinese patent medicine | no | unclear | unclear | yes | no | unclear |  |
| 2008 | Xiong YANHAO | | Jiangu Granule | | Calcium preparation | Chinese patent medicine | yes | unclear | yes | yes | no | unclear |  |
| 1998 | Wang XIAOYUN | | Bushen Yigu Ointment | | Blank | Chinese patent medicine | unclear | unclear | unclear | yes | no | unclear |  |
| 1997 | Feng SHILUN | | Zhibi Bugudan | | Longmu Zhuanggu Powder | Chinese patent medicine | unclear | unclear | unclear | yes | no | unclear |  |
| 2001 | Chen YAQIONG | | Jiarong Tablet | | Western medicine | Chinese patent medicine | unclear | unclear | unclear | yes | no | unclear |  |
| 2019 | Xie BAOHUA | | Zuogui Pill | | Blank | Chinese patent medicine | unclear | unclear | unclear | yes | no | unclear |  |
| 2012 | Liu SHUSHENG | | Lurong Zhuanggu Capsule | | Calcium preparation | Chinese patent medicine | no | unclear | unclear | yes | no | unclear |  |
| 2017 | Wang YONGJUN | | Shuimi Pill | | Western medicine | Chinese patent medicine | no | unclear | unclear | yes | no | yes |  |
| 2001 | Zhang GUANGMEI | | Xiangu Ning | | Western medicine | Chinese patent medicine | no | unclear | unclear | yes | no | yes |  |
| **Traditional Chinese medicin-RCT** | | | | | | | | | | | | | |
| **Year** | | **Author** | **Formula** | **Control** | | **Intervention Type** | **Random Sequence** | **Allocation Concealment** | **Blinding** | **Data Integrity** | **Selective Reporting** | **Other Bias** | |
| 2019 | | Cai Rongqi | Erxian Decoction | Western medicine | | Chinese decoction + Western medicine | Yes | Unclear | Unclear | Yes | Yes | Unclear | |
| 2023 | | Luo Kunling | Bushen Jianpi Formula | Calcium carbonate | | Decoction | Yes | Unclear | Unclear | Yes | Yes | Unclear | |
| 2023 | | Tang Zhili | Gushu Kang | Calcium preparation | | Decoction | Unclear | Unclear | Unclear | Yes | Yes | Unclear | |
| 2023 | | Li Dongping | Bushen Tongbi Decoction | Western medicine | | Decoction | Yes | Unclear | Unclear | Yes | Yes | Unclear | |
| 2023 | | Wang Shenghao | Gushu Formula | Western medicine | | Decoction | Yes | Unclear | Yes | Yes | Yes | Unclear | |
| 2023 | | Yang Ping | Zhuanggu Decoction | Western medicine | | Decoction | Yes | Unclear | Unclear | Yes | Yes | Unclear | |
| 2022 | | Zhang Yi | Bushen Shengxue Decoction | Western medicine | | Decoction | Unclear | Unclear | Unclear | Yes | Yes | Unclear | |
| 2022 | | Zhong Faming | Bushen Formula | Western medicine | | Decoction | Unclear | Unclear | Unclear | Yes | Yes | Unclear | |
| 2023 | | Chen Tianpeng | Qianggu Yin | Western medicine | | Decoction | Yes | Unclear | Unclear | Yes | No | Unclear | |
| 2022 | | Yin Xiaofei | Yishen Huoxue Formula | Surgery | | Chinese decoction + Western medicine | Unclear | Unclear | Unclear | Yes | No | Unclear | |
| 2022 | | Chen Yongxi | Bugan Qiangshen Zhuanggu Formula | Heat therapy | | Decoction | Unclear | Unclear | Unclear | Yes | No | Unclear | |
| 2021 | | Guo Jianbang | Chinese Herbal Formula | Western medicine | | Decoction | Yes | Unclear | Unclear | Yes | Unclear | Unclear | |
| 2021 | | Guo Xiaoli | Bushen Yigu Formula | Western medicine | | Decoction | Yes | Unclear | Unclear | Yes | Unclear | Unclear | |
| 2021 | | He Caijian | Yiqi Wenjing Formula | Western medicine | | Chinese decoction + Western medicine | Yes | Unclear | Unclear | Yes | Unclear | Yes | |
| 2021 | | Li Shaohua | Qianggu Yin | Western medicine | | Chinese decoction + Western medicine | Unclear | Unclear | Unclear | Yes | Unclear | Yes | |
| 2021 | | Cheng Yi | Yangxue Gushen Decoction | Western medicine | | Chinese decoction + Western medicine | Yes | Unclear | Unclear | Yes | Unclear | Unclear | |
| 2020 | | Gong Yunzhao | Guilu Erxian Decoction | Western medicine | | Chinese decoction + Western medicine | Yes | Unclear | Unclear | Yes | Unclear | Unclear | |
| 2020 | | Zhu Chuncheng | Yangxue Gushen Decoction | Western medicine | | Chinese decoction + Western medicine | Yes | Unclear | Unclear | Yes | Unclear | Unclear | |
| 2020 | | Wu Junqi | Ziyin Bushui Decoction | Western medicine | | Chinese decoction + Western medicine | Unclear | Unclear | Unclear | Yes | No | Yes | |
| 2020 | | Wang Hongqiao | Bushen Zhigan Formula | Surgery | | Decoction | Yes | Unclear | Unclear | Yes | Yes | Unclear | |
| 2020 | | Tang Xiaolu | Bushen Huoxue Formula | Western medicine | | Decoction | Unclear | Unclear | Unclear | Yes | No | Unclear | |
| 2020 | | Chen Yongxi | Bugan Qiangshen Zhuanggu Formula | Western medicine | | Chinese decoction + Western medicine | Unclear | Unclear | Unclear | No | Yes | Unclear | |
| 2020 | | Ju Yang | Erxian Decoction | Western medicine | | Chinese decoction + Western medicine | Unclear | Unclear | Unclear | Yes | Unclear | Unclear | |
| 2020 | | Zheng Xiuxia | Self-made Bushen Yangxue Huoxue ZhenTong Decoction | Western medicine | | Chinese decoction + fumigation | Unclear | Unclear | Unclear | Yes | Yes | Unclear | |
| 2019 | | Chen Limei | Bugugu Qiangjin Formula | Western medicine | | Chinese decoction + Western medicine | Yes | Unclear | Unclear | Yes | No | Unclear | |
| 2019 | | Xu Riming | Wenshen Gushu Formula | Western medicine | | Chinese decoction + Western medicine | Yes | Unclear | Unclear | Yes | No | Unclear | |
| 2019 | | Wang Chuanzhen | Bushen Zhuanggu Decoction | Western medicine | | Chinese decoction + Western medicine | Unclear | Unclear | Unclear | No | Yes | Unclear | |
| 2019 | | Wang Xiaowei | Bushen Huoxue Decoction | Calcium preparation | | Decoction | Yes | Unclear | Unclear | Yes | No | Unclear | |
| 2018 | | Huang Fuwei | Gushong Jiangu Formula | Qigu Capsule | | Decoction | Unclear | Unclear | Unclear | No | No | Unclear | |
| 2018 | | Wang Maopeng | Bushen Huoxue Decoction | Western medicine | | Chinese decoction + Western medicine | Yes | Unclear | Unclear | Yes | No | Unclear | |
| 2018 | | Chai Shengteng | Gukang Formula | Western medicine | | Decoction | Unclear | Unclear | Unclear | Yes | No | Unclear | |
| 2018 | | Wang Wang | Gengnian Jianshen Decoction | Xianling Gubao Capsule | | Decoction | Yes | Unclear | Unclear | Yes | No | Unclear | |
| 2018 | | Dong Chongyang | Jiawei Guilu Yanggu Decoction | Western medicine | | Chinese decoction + Western medicine | Yes | Unclear | Unclear | Yes | No | Unclear | |
| 2018 | | Qimeige | Bushen Tiaochong Formula | Western medicine | | Decoction | Unclear | Unclear | Unclear | Yes | No | Unclear | |
| 2018 | | Huang Huayong | Bushen Jianpi Formula | Western medicine | | Chinese decoction + Western medicine | No | Unclear | Unclear | Yes | No | Unclear | |
| 2017 | | Xie Lili | Yigu Decoction | Western medicine | | Chinese decoction + Western medicine | Unclear | Unclear | Unclear | Yes | No | Unclear | |
| 2017 | | Zhang Baoxin | Self-made Formula | Calcium preparation | | Decoction | Unclear | Unclear | Unclear | No | Yes | Unclear | |
| 2017 | | Li Jianjun | Erxian Decoction | Western medicine | | Chinese decoction + Western medicine | Unclear | Unclear | Unclear | Yes | No | Unclear | |
| 2017 | | Guo Xia | Bushen Huoxue Chinese Medicine | Western medicine | | Chinese decoction + Western medicine | Unclear | Unclear | Unclear | No | Yes | Unclear | |
| 2017 | | Keyimu Basiti | Bushen Tiaochong Formula | Western medicine | | Chinese decoction + Western medicine | Yes | Unclear | Unclear | Yes | No | Unclear | |
| 2017 | | Liu Ying | Bushen Huoxue Formula | Western medicine | | Decoction | Unclear | Unclear | Unclear | Yes | No | Unclear | |
| 2017 | | Chang Libo | Bugan Tiaoshen Decoction | Western medicine | | Decoction | Unclear | Unclear | Unclear | No | No | Unclear | |
| 2017 | | Huang Lehui | Duhuo Jisheng Decoction | Western medicine | | Decoction | Yes | Unclear | Unclear | Yes | No | Unclear | |
| 2017 | | Huang Qinghui | Siwu Decoction | Western medicine | | Chinese decoction + Western medicine | Unclear | Unclear | Unclear | Yes | No | Unclear | |
| 2017 | | Wang Shaofeng | Duhuo Jisheng Decoction | Western medicine | | Chinese decoction + Western medicine | No | Unclear | Unclear | Yes | No | Unclear | |
| 2017 | | Wu Chen | Duhuo Jisheng Decoction | Calcium preparation | | Decoction | Unclear | Unclear | Unclear | Yes | No | Unclear | |
| 2016 | | Zhan Lei | Bushen Huoxue Chinese Medicine | Calcium preparation | | Decoction | Unclear | Unclear | Unclear | Yes | No | Unclear | |
| 2016 | | Zou Laiyong | Jianpi Huoxue Bushen Formula | Western medicine | | Decoction | Unclear | Unclear | Unclear | Yes | No | Unclear | |
| 2016 | | Guo Qiang | Zibu Ganshen Medicine | Calcium preparation | | Decoction | Unclear | Unclear | Unclear | Yes | No | Unclear | |
| 2016 | | Chen Shunxian | Zibu Ganshen Zhuanggu Formula | Western medicine | | Chinese decoction + Western medicine | Unclear | Unclear | Unclear | Yes | No | Unclear | |
| 2015 | | Guo Ning | Gushu Kang | Western medicine | | Decoction | Unclear | Unclear | Unclear | Yes | Yes | Unclear | |
| 2015 | | Zhong Qiusheng | Jianpi Yiqi Formula | Western medicine | | Decoction | Unclear | Unclear | Unclear | Yes | No | Unclear | |
| 2015 | | Han Yan | Bushen Huoxue Formula | Western medicine | | Chinese decoction + Western medicine | Yes | Unclear | Unclear | Yes | No | Unclear | |
| 2015 | | Zhang Lixin | Gushusan | Western medicine | | Decoction | Yes | Unclear | Unclear | Yes | No | Unclear | |
| 2014 | | Zhang Xiaoling | Huayu Tianjing Formula | Western medicine | | Chinese decoction + Western medicine | Yes | Unclear | Unclear | Yes | No | Unclear | |
| 2014 | | Zhang Lixin | Self-made Gushusan | Western medicine | | Decoction | Unclear | Unclear | Unclear | No | No | Unclear | |
| 2014 | | Wang Yan | Duhuo Jisheng Decoction | Western medicine | | Chinese decoction + Western medicine | Unclear | Unclear | Unclear | No | No | Unclear | |
| 2013 | | Zhang Risong | Bushen Zhuanggu Decoction | Western medicine | | Decoction | No | Unclear | Unclear | Yes | No | Unclear | |
| 2022 | | Ning Jinyue | Self-made Zhuangshen Bugugu Formula | Western medicine | | Chinese decoction + Western medicine | Yes | Unclear | Unclear | Yes | No | Unclear | |
| 2022 | | Wang Lixin | Chai Ge Jieji Decoction | Western medicine | | Chinese decoction + Western medicine | Unclear | Unclear | Unclear | Yes | No | Unclear | |
| 2016 | | Fu Yuling | Shugan Zishen Huoxue Decoction | Western medicine | | Chinese decoction + Western medicine | Unclear | Unclear | Unclear | Yes | No | Unclear | |
| 2007 | | Zhang Yinping | Shigu Yin | Calcium preparation | | Decoction | Unclear | Unclear | Unclear | Yes | No | Unclear | |
| 2007 | | Li Zhen | Self-made Formula | Western medicine | | Chinese decoction + Western medicine | Unclear | Unclear | Unclear | Yes | No | Unclear | |
| 2012 | | Bai Jie | Gushu Kang | Calcium preparation | | Chinese decoction + Western medicine | Unclear | Unclear | Unclear | Yes | No | Unclear | |
| 2012 | | Zhao Ling | Self-made Qianggu Yin | Calcium preparation | | Decoction | Unclear | Unclear | Unclear | Yes | No | Unclear | |
| 2009 | | Cao Wei | Self-made Formula | Western medicine | | Chinese decoction + Western medicine | Unclear | Unclear | Unclear | Yes | No | Unclear | |
| 2005 | | Ke Qing | Self-made Jiangu Formula | Western medicine | | Decoction | Unclear | Unclear | Unclear | Yes | No | Unclear | |
| 2004 | | Liao Lin | Bushen Shengsui Decoction | Western medicine | | Decoction | Unclear | Unclear | Unclear | Yes | No | Unclear | |
| 2004 | | Xiang Keming | Duhuo Jisheng Decoction | Western medicine | | Chinese decoction + Western medicine | Unclear | Unclear | Unclear | Yes | No | Unclear | |
| 2013 | | Chen Jizheng | Chinese Herbal Formula | Blank | | Decoction | Unclear | Unclear | Unclear | Yes | No | Yes | |
| 2013 | | Diao Weixia | Yangxue Gushen Decoction | Western medicine | | Chinese decoction + Western medicine | Unclear | Unclear | Unclear | Yes | No | Yes | |
| 2013 | | Zhu Qingao | Erxian Decoction | Western medicine | | Chinese decoction + Western medicine | Yes | Unclear | Unclear | Yes | No | Yes | |
| 2012 | | Li Hanwen | Bushen Zhuanggu Formula | Western medicine | | Chinese decoction + Western medicine | Unclear | Unclear | Unclear | Yes | No | Yes | |
| 2011 | | Han Yan | Bushen Huoxue Formula | Western medicine | | Chinese decoction + Western medicine | Unclear | Unclear | Unclear | Yes | No | Unclear | |
| 2011 | | Chen Shuqing | Gushen Jianpi Formula | Calcium preparation | | Decoction | Unclear | Unclear | Unclear | Yes | No | Unclear | |
| 2011 | | Yang Linzhi | Yishen Qiangdu Huoxue Formula | Calcium preparation | | Decoction | No | Unclear | Unclear | No | Yes | Yes | |
| 2010 | | Chen Lina | Wenshen Yugong Decoction | Western medicine | | Chinese decoction + Western medicine | Unclear | Unclear | Unclear | Yes | No | Yes | |
| 2009 | | Lin Yifeng | Gukang | Western medicine | | Decoction | Unclear | Unclear | Unclear | Yes | No | Yes | |
| 2008 | | Wang Jiamou | Gukang | Western medicine | | Decoction | Unclear | Unclear | Unclear | Yes | No | Unclear | |
| 2008 | | Qiu Renbin | Jiangu Formula | Western medicine | | Decoction | Unclear | Unclear | Unclear | Yes | No | Unclear | |
| 2008 | | Huang Hongxing | Gukang | Western medicine | | Decoction | Unclear | Unclear | Unclear | Yes | No | No | |
| 2008 | | Luo Xiaoling | Jianpi Bushen Formula | Longmu Zhuanggu Chongji | | Decoction | Unclear | Unclear | Unclear | Yes | No | Unclear | |
| 2007 | | Han Xiangli | Shugan Zishen Huoxue Decoction | Western medicine | | Chinese decoction + Western medicine | Unclear | Unclear | Unclear | No | Yes | Unclear | |
| 2006 | | Liu Yinghua | Yiqi Jianpi Huoxue Bushen Formula | Western medicine | | Chinese decoction + Western medicine | Unclear | Unclear | Unclear | Yes | No | Unclear | |
| 2004 | | Zhong Ziru | Gushu Decoction | Longmu Zhuanggu Chongji | | Decoction | Unclear | Unclear | Unclear | No | Yes | Unclear | |
| 2003 | | Zhao Gang | Zishen Formula | Western medicine | | Chinese decoction + Western medicine | Unclear | Unclear | Unclear | Yes | No | Unclear | |
| 2002 | | Chen Fasheng | Self-made Gushen Decoction | Western medicine | | Chinese decoction + Western medicine | Unclear | Unclear | Unclear | Yes | No | Unclear | |
| 1999 | | Liang Keyu | Zenggu Decoction Sequential Therapy | Empirical formula | | Decoction | Yes | Unclear | Yes | Yes | No | No | |
| 1999 | | Deng Weimin | Bushen Zhuanggu Decoction | Western medicine | | Decoction | Unclear | Unclear | Unclear | Yes | No | Unclear | |
| 1997 | | Deng Weimin | Bushen Chinese Medicine | Western medicine | | Decoction | Unclear | Unclear | Unclear | Yes | No | Unclear | |
| 2022 | | Jiang Yihua | Bushen Jiangu Decoction | Calcium preparation | | Decoction | No | Unclear | Unclear | Yes | No | Unclear | |
| 2008 | | Tu Pingsheng | Shuanggu Decoction | Blank | | Decoction | Unclear | Unclear | Unclear | Yes | No | Unclear | |
| 2014 | | Li Chunwen | Qianggu Yin | Western medicine | | Chinese decoction + Western medicine | Unclear | Unclear | Unclear | Yes | No | No | |
| 2017 | | Li Caiyi | Gushu Kang | Western medicine | | Chinese decoction + Western medicine | No | Unclear | Unclear | Yes | No | Unclear | |
| 2007 | | Zeng Wuxiong | Bushen Huoxue Compound | Western medicine | | Decoction | Yes | Unclear | Yes | Yes | No | Unclear | |
| 2017 | | Chen Guilin | Chinese Medicine | Western medicine | | Chinese decoction + Western medicine | Unclear | Unclear | Unclear | Yes | No | Unclear | |
| 2012 | | Chen Shuqing | Gushen Jianpi Formula | Calcium preparation | | Decoction | Unclear | Unclear | Unclear | Yes | No | Unclear | |
| 2008 | | Dong Shulin | Chinese Medicine | Western medicine | | Chinese decoction + Western medicine | Unclear | Unclear | Unclear | Yes | No | Unclear | |
| 2010 | | Xu Xuemei | Self-made Gushongyu Decoction | Qianggu Capsule | | Decoction | Unclear | Unclear | Unclear | Yes | No | Unclear | |
| 2014 | | Zhang Lixin | Self-made Gushusan | Western medicine | | Decoction | Unclear | Unclear | Unclear | Yes | No | Unclear | |
| 2017 | | Fan Shuai | Chinese Medicine | Western medicine | | Decoction | Yes | Unclear | Unclear | Yes | No | Unclear | |
| 2010 | | Deng Wei | Self-made Jianpi Yishen Qingre Formula | Calcium preparation | | Decoction | Unclear | Unclear | Unclear | Yes | No | No | |
| 2011 | | Li Zhongping | Kanggusong Decoction | Calcium preparation | | Decoction | Unclear | Unclear | Unclear | Yes | No | Unclear | |
| 2017 | | Dong Chongyang | Bushen Zhuanggu Decoction | Western medicine | | Chinese decoction + Western medicine | Yes | Unclear | Yes | Yes | No | Unclear | |
| 2012 | | Lao Ruiping | Bushen Jianpi Huoxue Formula | Calcium preparation | | Decoction | Unclear | Unclear | Unclear | Yes | No | Unclear | |
| 2017 | | Zhang Penggui | Duhuo Jisheng Decoction | Western medicine | | Chinese decoction + Western medicine | Unclear | Unclear | Unclear | Yes | No | Unclear | |
| 2017 | | Liu Xiaofang | Chinese Medicine | Western medicine | | Decoction | No | Unclear | Unclear | Yes | No | Unclear | |
| 2000 | | An Shengjun | Bushen Chinese Medicine | Western medicine | | Decoction | Yes | Unclear | Yes | Yes | No | No | |
| 1996 | | Li Longmin | Tanshinone | Western medicine | | Decoction | No | Unclear | Unclear | No | No | Yes | |
| 2001 | | Xie Jing | Bushen Chinese Medicine | Calcium preparation | | Decoction | Unclear | Unclear | Unclear | Yes | No | Unclear | |
| 2008 | | Tang Ling | Gengnian Kang | Western medicine | | Chinese decoction + Western medicine | No | Unclear | Unclear | No | Yes | Unclear | |
| 2016 | | Cai Rongqi | Erxian Decoction | Western medicine | | Chinese decoction + Western medicine | No | Unclear | Unclear | Yes | No | Unclear | |
| 2009 | | Li Yi | Yigu Decoction | Calcium preparation | | Decoction | Unclear | Unclear | Unclear | Yes | No | Unclear | |
| 2011 | | Lin Xiaosheng | Shugan Yishen Decoction | Western medicine | | Decoction | Unclear | Unclear | Unclear | Yes | No | Unclear | |
| 2011 | | Sun Guihe | Chinese Medicine + Nursing | Chinese Medicine | | Decoction | Unclear | Unclear | Unclear | Yes | No | Unclear | |
| 2007 | | Zhang, Ge | Epimedium (Yinyanghuo) | Blank | | Decoction | Unclear | Unclear | Unclear | Yes | No | Unclear | |
| 2017 | | Shi, Zhen-Yu | Qianggu Yin | Blank | | Decoction | Unclear | Unclear | Unclear | Yes | No | Unclear | |

| **Other Chinese medicine-RCT** | | | | | | | | | | |
| --- | --- | --- | --- | --- | --- | --- | --- | --- | --- | --- |
| **Year** | **Author** | **Intervention** | **Control** | **Intervention Type** | **Random Sequence** | **Allocation Concealment** | **Blinding** | **Data Completeness** | **Selective Reporting** | **Other Biases** |
| 2024 | Pan Yanlu | Chinese herbal fumigation | Conventional Western medicine | Fumigation | Yes | Unclear | Unclear | Yes | No | Unclear |
| 2023 | Li Jianzhu | Acupuncture combined with herbal application | — | Chinese + Chinese | Yes | Unclear | Unclear | Yes | No | Unclear |
| 2023 | Huang Kui | Bushen Huoxue Decoction | Western medicine | Chinese + Western medicine | Yes | Unclear | Unclear | Yes | No | Unclear |
| 2022 | Li Shaohua | Acupoint application | Western medicine | Acupoint application | Yes | Unclear | Unclear | Yes | No | Unclear |
| 2021 | Wang Jinhua | Kudzu root extract | Western medicine | Chinese + Western medicine | Unclear | Unclear | Unclear | Yes | No | Unclear |
| 2020 | Li Qi | Modified Wuqinxi exercise | Wuqinxi | Functional exercise | Unclear | Unclear | Unclear | Yes | No | Unclear |
| 2019 | Luo Yong | Herbal fumigation combined with salmon calcitonin | Western medicine | Fumigation + Western medicine | Yes | Unclear | Unclear | Yes | No | Unclear |
| 2019 | Zhao Jun | Integrated TCM regimen | Western medicine | Chinese + Western medicine | Yes | Unclear | Unclear | Yes | No | Unclear |
| 2018 | Xu Feng | Chinese medicine | Western medicine | Chinese + Western medicine | Unclear | Unclear | Unclear | Yes | No | Unclear |
| 2018 | Chen Wenliang | Yiqi Wenjing Formula combined with modified Wuqinxi | Blank control | Chinese + Chinese | Unclear | Unclear | Unclear | Yes | No | Unclear |
| 2018 | Wu Qimei | Moxibustion at Shenque acupoint | Calcium preparation | Acupuncture | Yes | Unclear | Yes | Yes | No | Unclear |
| 2017 | Ma Junyi | Acupoint application therapy | Western medicine | Acupoint + Western medicine | Unclear | Unclear | Unclear | Yes | No | Unclear |
| 2017 | Li Aiqing | Bushen Huoxue Decoction + Du moxibustion | Du moxibustion | Chinese + Chinese | Unclear | Unclear | Unclear | Yes | No | Unclear |
| 2015 | He Kanghong | Acupoint herbal application | Calcium preparation | Acupoint application | Unclear | Unclear | Unclear | Yes | No | Unclear |
| 2015 | Liu Hanjiao | Bushen Huoxue Formula application | Western medicine | Acupoint application | Unclear | Unclear | Unclear | Yes | No | Unclear |
| 2015 | Chen Jie | Herbal diet combined with Baduanjin exercise | Baduanjin | Chinese + Chinese | Unclear | Unclear | Unclear | Yes | No | Unclear |
| 2014 | Du Xiaoli | Herbal diet combined with Baduanjin exercise | Western medicine | Chinese + Chinese | Unclear | Unclear | Unclear | Yes | No | Unclear |
| 2013 | Huang Ke | Acupuncture combined with ultrasound therapy for lumbar pain | Western medicine | Chinese + Western medicine | Unclear | Unclear | Unclear | Yes | No | Unclear |
| 2016 | Wang Na | Herbal paste + fire needle therapy | Calcium preparation | Chinese + Chinese | Unclear | Unclear | Unclear | Yes | No | Unclear |
| 2011 | Li Cuiqin | Bushen Huoxue Decoction combined with acupuncture | Western medicine | Chinese + Chinese | Unclear | Unclear | Unclear | Yes | No | Unclear |
| 2012 | Ouyang Sui | Integrated TCM and Western medicine effect on cytokines in perimenopausal osteoporosis | Western medicine | Chinese + Western medicine | Unclear | Unclear | Unclear | Yes | No | Unclear |
| 2009 | Wang Jianfa | Acupuncture therapy | Western medicine | Acupuncture | Unclear | Unclear | Unclear | Yes | No | Unclear |
| 2005 | Zhang Yurong | Chinese medicine + massage | Calcium preparation | Chinese + Chinese | Unclear | Unclear | Unclear | Yes | No | Unclear |
| 2024 | Li Lili | Internal and external Chinese medicine combined with Western therapy | Western medicine | Chinese + Western medicine | Yes | Unclear | Unclear | Yes | No | Unclear |
| 2023 | Huang Zhihui | Chinese herbal fumigation | Western medicine | Fumigation + Western medicine | Yes | Unclear | Unclear | Yes | No | Unclear |
| 2024 | Huang Yurong | Wax therapy combined with health education | Health education | Wax therapy | Yes | Unclear | Unclear | Yes | No | Unclear |
| 2003 | Pan Wenyu | Fire needle acupuncture | Chinese medicine | Acupuncture | Unclear | Unclear | Unclear | Yes | No | Unclear |
| 2012 | Li Guoying | Acupuncture combined with Guizhi Fuling Decoction | Western medicine | Chinese + Western medicine | Unclear | Unclear | Unclear | Yes | No | Unclear |
| 2011 | Dong Guifang | Integrated TCM and Western medicine for perimenopausal osteoporosis | Calcium preparation | Chinese + Western medicine | Unclear | Unclear | Unclear | Yes | No | Yes |
| 2020 | Ren Z.Q. | Overall Adjustment Acupuncture for PMOP: Randomized Sham-Controlled Trial | Blank | Acupuncture | Yes | Unclear | Yes | Yes | No | No |
| 2022 | Chen Y.T. | Lingnan Chen’s Acupuncture for PMOP and Serum GH/IGF-1 | Blank | Acupuncture | Unclear | Unclear | Unclear | Yes | No | Unclear |
| 2020 | Chen Guizhen | Biomarkers of PMOP and Mechanism of Catgut Embedding in Acupoints | Western medicine | Thread embedding | Yes | Unclear | Yes | Yes | No | No |
| 2022 | Lu Lingyun | Moxibustion as Adjuvant Therapy for Preventing Bone Loss in PMOP | Calcium preparation | Acupuncture | Unclear | Unclear | Unclear | Yes | No | Unclear |
| 2015 | Liu Baoxi | Effect of Modified Eight-Section Brocade (Baduanjin) on PMOP | Calcium preparation | Functional exercise | Unclear | Unclear | Unclear | Yes | No | Unclear |

| **Meta-analysis Quality Evaluation Form** | | | | | | | | | | | | | |
| --- | --- | --- | --- | --- | --- | --- | --- | --- | --- | --- | --- | --- | --- |
| **Year** | **Author** | **PICO** | **Research Method** | **Study Type** | **Search Strategy** | **Dual Screening** | **Dual Data Extraction** | **Risk of Bias Assessment** | **Quantitative Synthesis** | **Results Discussion** | **Funding Information** | **All Results Reported** | **Total Score** |
| 2022 | Xiang, Kemeng | 1 | 1 | 1 | 1 | 1 | 1 | 1 | 1 | 1 | 1 | 1 | 11 |
| 2017 | Jin, Yong-Xiang | 1 | 1 | 1 | 1 | 1 | 1 | 1 | 1 | 1 | 0 | 1 | 10 |
| 2017 | Zhang, Yili | 1 | 1 | 1 | 1 | 1 | 1 | 1 | 1 | 1 | Unclear | 1 | 10 |
| 2020 | Si, Yuhao | 1 | 1 | 1 | 1 | 1 | 1 | 1 | 0 | 1 | 1 | 1 | 10 |
| 2021 | Lou, Fanglian | 1 | 1 | 1 | 1 | 1 | 1 | 1 | 1 | 1 | 0 | 1 | 10 |
| 2022 | Luo, Minghui | 1 | 0 | 1 | 1 | 1 | 1 | 1 | 1 | 1 | 1 | 1 | 10 |
| 2022 | Zhao, Jinlong | 1 | 1 | 1 | 1 | 1 | 1 | 1 | 0 | 1 | 1 | 1 | 10 |
| 2024 | Han, Yuxin | 1 | 1 | 1 | 1 | 1 | 1 | 1 | 1 | 1 | 0 | 1 | 10 |
| 2019 | Huang, Fan | 0 | 1 | 1 | 1 | 1 | 1 | 1 | Unclear | 1 | 1 | 1 | 9 |
| 2019 | Zhao, Siyi | 1 | Unclear | 1 | 1 | 1 | 1 | 1 | 1 | 1 | Unclear | 1 | 9 |
| 2020 | An, Hongqiang | 1 | 1 | 1 | 1 | 1 | 1 | 1 | Unclear | 1 | 0 | 1 | 9 |
| 2020 | Ling, Qiuling | 1 | Unclear | 1 | 1 | 1 | 1 | 1 | 1 | 1 | Unclear | 1 | 9 |
| 2020 | Wang, Yang | 1 | Unclear | 1 | 1 | 1 | 1 | 1 | 1 | 1 | Unclear | 1 | 9 |
| 2021 | Chen, Pei | 1 | Unclear | 1 | 1 | 1 | 1 | 1 | 1 | 1 | Unclear | 1 | 9 |
| 2022 | Cai, Xinyu | 1 | Unclear | 1 | 1 | 1 | 1 | 1 | 1 | 1 | Unclear | 1 | 9 |
| 2019 | Huang, Weiwei | 1 | 1 | 1 | 1 | Unclear | Unclear | 1 | 1 | 1 | Unclear | 1 | 8 |
| 2021 | Wang, Qingying | 1 | Unclear | 1 | 1 | 1 | Unclear | 1 | 1 | 1 | Unclear | 1 | 8 |
| 2005 | Xie, Yanming | 1 | 1 | 1 | 0 | 1 | Unclear | 1 | Unclear | 1 | Unclear | 1 | 7 |
| 2008 | Huang, Xuchun | 1 | 0 | 1 | Unclear | 1 | 1 | 0 | 1 | 1 | Unclear | 1 | 7 |
| 2019 | An, Yifang | 1 | Unclear | 1 | 1 | Unclear | Unclear | 1 | 1 | 1 | Unclear | 1 | 7 |
| 2021 | Jin, Yongxiang | 1 | 1 | 1 | 1 | 1 | 1 | 1 | 1 | 1 | 0 | 1 | 10 |
| 2020 | Chen, Taihong | 1 | Unclear | 1 | 1 | Unclear | Unclear | Unclear | 1 | 1 | Unclear | 1 | 6 |
| 2005 | Liu, Yu | 1 | 0 | 1 | Unclear | Unclear | Unclear | Unclear | 1 | 1 | Unclear | 1 | 5 |
| 2022 | Wang, Jing | N | Unclear | 1 | 1 | Unclear | Unclear | 1 | 0 | 1 | Unclear | 1 | 5 |
| 2004 | Chen, Li | 1 | Unclear | 1 | Unclear | Unclear | Unclear | Unclear | 0 | 1 | Unclear | 1 | 4 |

| **Clinical Guideline Quality Evaluation Form** | | | | | | | | | | | | | |
| --- | --- | --- | --- | --- | --- | --- | --- | --- | --- | --- | --- | --- | --- |
| **Main Domain** | **Sub-item** | **Guideline for TCM Clinical Practice of Postmenopausal Osteoporosis** | | | | | | **Guideline for TCM Diagnosis and Treatment of Postmenopausal Osteoporosis (2019 Edition)** | | | | | |
|  |  | **Score 1** | | **Score 2** | | **Total** | **ICC (%)** | **Score 1** | | **Score 2** | | **Total** | **ICC (%)** |
| **Scope and Purpose** | Objective | 6 | 17 | 6 | 16 | 33 | 91.67 | 6 | 16 | 6 | 18 | 34 | 94.55 |
|  | Clinical Question | 6 |  | 6 |  |  |  | 6 |  | 6 |  |  |  |
|  | Target Population | 5 |  | 4 |  |  |  | 4 |  | 6 |  |  |  |
| **Stakeholder Involvement** | Involvement of Experts | 6 | 17 | 5 | 15 | 32 | 88.89 | 5 | 15 | 5 | 16 | 31 | 86.11 |
|  | Target Users’ Viewpoints | 7 |  | 6 |  |  |  | 7 |  | 6 |  |  |  |
|  | Applicability Definition | 4 |  | 4 |  |  |  | 3 |  | 5 |  |  |  |
| **Rigor of Development** | Systematic Method | 6 | 36 | 5 | 33 | 69 | 82.14 | 5 | 35 | 5 | 35 | 70 | 83.33 |
|  | Evidence Standards | 7 |  | 6 |  |  |  | 6 |  | 6 |  |  |  |
|  | Strengths & Limitations | 5 |  | 4 |  |  |  | 5 |  | 5 |  |  |  |
|  | Formulation Method | 5 |  | 5 |  |  |  | 5 |  | 4 |  |  |  |
|  | Consideration of Opinions | 6 |  | 4 |  |  |  | 6 |  | 6 |  |  |  |
|  | Link between Evidence & Recommendations | 3 |  | 3 |  |  |  | 3 |  | 3 |  |  |  |
|  | Peer Review | 3 |  | 4 |  |  |  | 4 |  | 4 |  |  |  |
|  | Updating Process | 1 |  | 2 |  |  |  | 1 |  | 2 |  |  |  |
| **Clarity of Presentation** | Clarity of Recommendations | 6 | 17 | 7 | 16 | 33 | 91.67 | 6 | 16 | 6 | 15 | 31 | 86.11 |
|  | Alternatives for Management | 6 |  | 5 |  |  |  | 6 |  | 5 |  |  |  |
|  | Easily Identifiable Key Points | 5 |  | 4 |  |  |  | 4 |  | 4 |  |  |  |
| **Applicability** | Barriers & Facilitators | 3 | 7 | 4 | 11 | 18 | 37.5 | 3 | 9 | 3 | 9 | 18 | 37.5 |
|  | Supporting Tools | 1 |  | 2 |  |  |  | 2 |  | 2 |  |  |  |
|  | Resource Implications | 1 |  | 2 |  |  |  | 2 |  | 2 |  |  |  |
|  | Monitoring / Audit Criteria | 2 |  | 3 |  |  |  | 2 |  | 2 |  |  |  |
| **Editorial Independence** | Funding Disclosure | 1 | 2 | 1 | 2 | 4 | 16.67 | 1 | 2 | 1 | 2 | 4 | 16.67 |
|  | Conflict of Interest | 1 |  | 1 |  |  |  | 1 |  | 1 |  |  |  |

| **Descriptive research Quality Evaluation Form** | | | | | | | | | | | | |
| --- | --- | --- | --- | --- | --- | --- | --- | --- | --- | --- | --- | --- |
| Year | Author | Inclusion Criteria | Diagnostic Certainty | Diagnostic Standard | Continuity | Comprehensiveness | Population | Clinical Design | Outcome | Geographic Coverage | Methodology | Score |
| 2023 | Huang, Hao-Qiang | 1 | 1 | 1 | 0 | 0 | 1 | 0 | 1 | 0 | 1 | 6 |
| 2023 | Liao, Yi-Tao | 1 | 1 | 1 | 0 | 0 | 1 | 0 | 1 | 0 | 0 | 5 |
| 2023 | Liu, Meng-Min | 1 | 1 | 1 | 1 | 0 | 1 | 0 | 1 | 0 | 1 | 7 |
| 2023 | Liu, Shu-Hua | 1 | 1 | 1 | 1 | 0 | 1 | 0 | 1 | 0 | 0 | 6 |
| 2023 | Peng, Hong-Cheng | 1 | 1 | 1 | 1 | 1 | 1 | 0 | 1 | 1 | 1 | 9 |
| 2023 | Xu, Zhen | 1 | 1 | 1 | 0 | 1 | 1 | 1 | 1 | 0 | 1 | 8 |
| 2022 | Wang, Jing | 0 | 1 | 0 | 1 | 0 | 1 | 1 | 1 | 1 | 1 | 7 |
| 2022 | Yuan, Jia-Yao | 1 | 0 | 1 | 1 | 0 | 1 | 1 | 1 | 0 | 1 | 7 |
| 2021 | Li, Jian-Yang | 1 | 1 | 1 | 1 | 1 | 1 | 1 | 1 | 0 | 1 | 9 |
| 2021 | Peng, Guo-Lan | 1 | 0 | 1 | 1 | 0 | 1 | 0 | 1 | 0 | 1 | 6 |
| 2021 | Xiao, Fei | 1 | 1 | 1 | 1 | 1 | 1 | 0 | 1 | 0 | 1 | 8 |
| 2020 | Xu, Bing | 1 | 1 | 1 | 1 | 0 | 1 | 0 | 1 | 0 | 1 | 7 |
| 2020 | Wang, Yan | 1 | 1 | 1 | 1 | 1 | 1 | 0 | 1 | 0 | 1 | 8 |
| 2020 | Miao, Ke-Tuan | 1 | 1 | 1 | 1 | 1 | 1 | 0 | 1 | 0 | 1 | 8 |
| 2020 | Li, Ming-Li | 0 | 0 | 1 | 1 | 0 | 0 | 0 | 1 | 0 | 0 | 3 |
| 2019 | Li, Jian-Yang | 1 | 1 | 1 | 1 | 1 | 1 | 1 | 1 | 1 | 1 | 10 |
| 2018 | Xiao, Ben-Hao | 1 | 1 | 1 | 1 | 1 | 1 | 0 | 1 | 0 | 1 | 8 |
| 2018 | Kang, Jian | 0 | 0 | 1 | 1 | 1 | 0 | 0 | 0 | 0 | 0 | 3 |
| 2017 | Sun, Shu-Ya | 1 | 1 | 0 | 1 | 0 | 1 | 0 | 1 | 0 | 1 | 6 |
| 2017 | Xu, Hui-Juan | 1 | 1 | 1 | 1 | 1 | 1 | 0 | 1 | 0 | 1 | 8 |
| 2016 | Xu, Hui-Juan | 1 | 1 | 1 | 1 | 1 | 1 | 0 | 1 | 0 | 1 | 8 |
| 2016 | Li, Sheng-Qiang | 1 | 1 | 1 | 1 | 1 | 1 | 0 | 1 | 1 | 1 | 9 |
| 2016 | Miao, Ying | 1 | 0 | 1 | 1 | 1 | 1 | 1 | 1 | 0 | 1 | 8 |
| 2016 | Sui, Cheng-Zhi | 1 | 1 | 1 | 1 | 1 | 0 | 1 | 1 | 0 | 1 | 8 |
| 2016 | Tang, Hong-Yu | 1 | 1 | 1 | 1 | 1 | 0 | 1 | 1 | 0 | 1 | 8 |
| 2015 | Wang, Dan | 0 | 1 | 1 | 0 | 1 | 0 | 1 | 1 | 0 | 0 | 5 |
| 2015 | Hu, Jin-Tao | 1 | 1 | 1 | 1 | 1 | 1 | 0 | 1 | 0 | 1 | 8 |
| 2014 | Du, Xiao-Li | 1 | 1 | 0 | 1 | 1 | 1 | 1 | 1 | 0 | 1 | 8 |
| 2014 | Zhang, Guo-Shan | 1 | 1 | 1 | 1 | 1 | 1 | 0 | 1 | 0 | 1 | 8 |
| 2013 | Zhang, Li-Ping | 1 | 0 | 1 | 1 | 1 | 1 | 0 | 1 | 0 | 0 | 6 |
| 2012 | Cheng, Zhi-An | 1 | 1 | 1 | 1 | 1 | 1 | 1 | 1 | 1 | 1 | 10 |
| 2011 | Nie, Da-Rong | 1 | 1 | 1 | 1 | 0 | 1 | 0 | 1 | 1 | 1 | 8 |
| 2011 | Qian, Yang-Pei-Juan | 1 | 1 | 1 | 1 | 0 | 1 | 0 | 1 | 0 | 1 | 7 |
| 2010 | Wang, Bin | 1 | 1 | 0 | 1 | 1 | 1 | 1 | 1 | 0 | 1 | 8 |
| 2010 | Wei, Bao-Jun | 1 | 1 | 1 | 1 | 0 | 1 | 0 | 1 | 0 | 1 | 7 |
| 2009 | Chai, Sheng-Ting | 1 | 1 | 0 | 1 | 1 | 1 | 1 | 1 | 0 | 1 | 8 |
| 2009 | Qian, Yang-Pei-Juan | 1 | 1 | 1 | 1 | 0 | 1 | 0 | 1 | 0 | 1 | 7 |
| 2008 | Zhang, Sui-Jian | 1 | 1 | 1 | 1 | 1 | 0 | 0 | 1 | 0 | 1 | 7 |
| 2008 | Lu, Li-Fang | 1 | 0 | 0 | 1 | 0 | 0 | 1 | 1 | 0 | 0 | 4 |
| 2008 | Zheng, Xin | 0 | 0 | 0 | 1 | 0 | 0 | 1 | 1 | 0 | 0 | 3 |
| 2008 | Cheng, Zhi-An | 1 | 0 | 0 | 1 | 0 | 1 | 1 | 1 | 0 | 0 | 5 |
| 2007 | Huang, Hong-Xing | 1 | 1 | 0 | 1 | 0 | 0 | 0 | 1 | 0 | 1 | 5 |
| 2006 | Lin, Yi-Feng | 1 | 1 | 0 | 1 | 1 | 1 | 1 | 1 | 0 | 1 | 8 |
| 2006 | Ge, Ji-Rong | 1 | 1 | 1 | 1 | 1 | 1 | 1 | 1 | 0 | 1 | 9 |
| 2006 | Du, Ying | 1 | 1 | 1 | 1 | 1 | 1 | 0 | 1 | 0 | 1 | 8 |
| 2005 | Li, Juan | 1 | 1 | 1 | 1 | 1 | 1 | 0 | 1 | 0 | 1 | 8 |
| 2005 | Ge, Ji-Rong | 1 | 0 | 1 | 1 | 0 | 1 | 1 | 1 | 0 | 1 | 7 |
| 2005 | Zou, Chong-Qi | 1 | 1 | 1 | 1 | 1 | 1 | 0 | 1 | 1 | 1 | 9 |
| 2004 | Deng, Wei-Min | 1 | 1 | 1 | 1 | 0 | 1 | 0 | 1 | 0 | 0 | 6 |
| 2004 | Ma, Quan | 0 | 0 | 1 | 1 | 1 | 1 | 0 | 1 | 0 | 0 | 5 |
| 2001 | Zhou, Zhi-Kun | 0 | 0 | 1 | 1 | 0 | 0 | 1 | 0 | 0 | 1 | 4 |
| 2001 | Song, Xian-Wen | 0 | 0 | 1 | 1 | 1 | 0 | 0 | 0 | 0 | 0 | 3 |
| 2000 | An, Shu | 1 | 0 | 1 | 0 | 1 | 1 | 0 | 1 | 0 | 0 | 5 |
| 2000 | Zhou, Pi-Qi | 1 | 0 | 1 | 1 | 1 | 0 | 1 | 1 | 0 | 1 | 7 |
| 2000 | Qi, Zhen-Xi | 1 | 1 | 0 | 1 | 1 | 1 | 1 | 1 | 0 | 1 | 8 |
| 1999 | Chen, Ying-Ying | 1 | 0 | 1 | 0 | 0 | 1 | 0 | 1 | 0 | 1 | 5 |
| 1999 | Cui, Tong-Hai | 1 | 0 | 0 | 1 | 1 | 1 | 0 | 1 | 0 | 0 | 5 |
| 1998 | Bian, Mei-Lu | 1 | 1 | 0 | 1 | 1 | 1 | 1 | 1 | 0 | 1 | 8 |
| 1997 | Liu, Qing-Si | 1 | 1 | 1 | 1 | 0 | 1 | 0 | 1 | 0 | 0 | 6 |
| 1995 | Zhang, Nai-Zheng | 1 | 0 | 0 | 1 | 0 | 0 | 1 | 1 | 0 | 0 | 4 |
